# The Role of Eosinophil-Derived Neurotoxin and Vascular Endothelial Growth Factor in the Pathogenesis of Eosinophilic Asthma

**DOI:** 10.3390/cells12091326

**Published:** 2023-05-06

**Authors:** Maciej Tota, Julia Łacwik, Julia Laska, Łukasz Sędek, Krzysztof Gomułka

**Affiliations:** 1 Student Scientific Group of Adult Allergology, Clinical Department of Internal Medicine, Pneumology and Allergology, Wroclaw Medical University, 50-369 Wrocław, Poland; 2 Student Scientific Group of Microbiology and Immunology, Department of Microbiology and Immunology, Zabrze, Medical University of Silesia in Katowice, 40-055 Katowice, Poland; 3 Department of Microbiology and Immunology, Zabrze, Medical University of Silesia in Katowice, 40-055 Katowice, Poland; 4 Clinical Department of Internal Medicine, Pneumology and Allergology, Wroclaw Medical University, 50-369 Wrocław, Poland

**Keywords:** asthma, VEGF, EDN, IL-4, IL-5, IL-13, anti-VEGF, anti-eosinophil, treatment, pathophysiology

## Abstract

Asthma is a chronic complex pulmonary disease characterized by airway inflammation, remodeling, and hyperresponsiveness. Vascular endothelial growth factor (VEGF) and eosinophil-derived neurotoxin (EDN) are two significant mediators involved in the pathophysiology of asthma. In asthma, VEGF and EDN levels are elevated and correlate with disease severity and airway hyperresponsiveness. Diversity in VEGF polymorphisms results in the variability of responses to glucocorticosteroids and leukotriene antagonist treatment. Targeting VEGF and eosinophils is a promising therapeutic approach for asthma. We identified lichochalcone A, bevacizumab, azithromycin (AZT), vitamin D, diosmetin, epigallocatechin gallate, IGFBP-3, Neovastat (AE-941), endostatin, PEDF, and melatonin as putative add-on drugs in asthma with anti-VEGF properties. Further studies and clinical trials are needed to evaluate the efficacy of those drugs. AZT reduces the exacerbation rate and may be considered in adults with persistent symptomatic asthma. However, the long-term effects of AZT on community microbial resistance require further investigation. Vitamin D supplementation may enhance corticosteroid responsiveness. Herein, anti-eosinophil drugs are reviewed. Among them are, e.g., anti-IL-5 (mepolizumab, reslizumab, and benralizumab), anti-IL-13 (lebrikizumab and tralokinumab), anti-IL-4 and anti-IL-13 (dupilumab), and anti-IgE (omalizumab) drugs. EDN over peripheral blood eosinophil count is recommended to monitor the asthma control status and to assess the efficacy of anti-IL-5 therapy in asthma.

## 1. Introduction

### 1.1. Eosinophilic Asthma

Asthma is one of the most common chronic pulmonary diseases and affects approximately 400 million people worldwide with a gradually increasing incidence [1]. In 2019, the global prevalence of asthma was 9.8%. It is estimated that over 260 million people have poorly controlled asthma [2,3]. Asthma is an inflammatory disease characterized by respiratory symptoms such as wheezing, chronic cough, chest tightness, and shortness of breath that results from bronchial hyperreactivity and inflammation [4,5]. Allergens, obesity, tobacco smoke, exercise, cold air, genetic mutations, and systemic eosinophilia are factors that induce chronic inflammation leading to airway hyperresponsiveness [6,7]. Chronic inflammation causes airway swelling, remodeling, and excessive mucus secretion. The airway remodeling process is driven by subepithelial fibrosis, increased smooth muscle mass, angiogenesis, and proliferation of mucosal glands, which result in irreversible structural changes [6,8].

Asthma is divided into allergic and non-allergic categories. Allergic asthma is triggered by allergens and is mainly attributed to abnormal T-helper type 2 inflammation. Non-allergic asthma is triggered by various factors such as aspirin, lung infection, exercise, cold, stress, and obesity [9,10,11]. Asthma can also be classified into neutrophilic (Th1 and Th17 response) and eosinophilic (Th2 response) [12].

Eosinophilic asthma (EA) is the clinical inflammatory phenotype with abundant levels of sputum, airway, and/or blood eosinophils. The European Academy of Allergy and Clinical Immunology (EAACI) defines EA as sputum eosinophil levels greater than 1%, an asthma-related peripheral blood eosinophil count of ≥150 cells/μL, or fractional exhaled nitric oxide (FeNO) of ≥20 ppb. Severe eosinophilic asthma is described as eosinophilic inflammation determined by a blood eosinophil level of either 300+ cells/μL in the last 12 months or 150+ cells/μL at the initiation [13]. Contrarily, noneosinophilic asthma may exhibit low eosinophil counts with elevated neutrophils, mixed granulocyte inflammatory cells, or only a small number of inflammatory cells, known as paucigranulocytic inflammation [14].

Eosinophils are white blood cells that defend against parasitic infections and contribute to the allergic inflammatory response [15]. These cells are recruited and activated in lung tissue as part of asthma’s pathophysiology. Evidence suggests that eosinophils contribute to airway dysfunction and tissue remodeling [16,17]. Eosinophils modulate the immune response by releasing mediators, proinflammatory cytokines, and toxic proteins from their cytoplasmic granularity [18]. Eosinophil granularity contains several essential proteins, including the major basic protein, eosinophil peroxidase, eosinophil-derived neurotoxin (EDN), and eosinophil cationic protein (ECP) [19].

### 1.2. Pathophysiology of Asthma

The pathophysiology of structural changes in the airways is mainly due to Th1, Th2, and Th17 dysregulation [6,20]. Abnormal T-helper type 2 (Th2) inflammation is the most important pathological process in asthma, mediated by Th2 cytokines such as interleukin (IL)-5, IL-4, and IL-13. Approximately 50% of mild to moderate asthma and a large percentage of severe asthma cases are induced by Th2-mediated inflammation [9,21,22].

#### 1.2.1. Th2 Response

Th2 cells and the cytokines they secrete (IL-4, IL-5, IL-13, and IL-9) are responsible for most of the pathological changes seen in asthma [23]. Once the sensitization phase has passed, the repeated arrival of the allergen in the lower airways results in the induction of mast cells by IgE to release mediators such as leukotrienes (LTs), histamine, and interleukins. These mediators irritate airway smooth muscle and induce bronchospasm [23,24,25]. In addition, IL-5 causes the production and chemotaxis of eosinophils in the lungs [26]. IL-13 sensitizes airway smooth muscle to spasm, stimulates epithelial cells to secrete mucus, and induces fibrosis [9,27].

Based on the state of Th2 inflammation, the disease can be divided into two groups: Th2-high and Th2-low asthma. Th2-high asthma is characterized by eosinophilic airway inflammation (eosinophilic asthma), which is associated with an increased number of eosinophils in the blood, while Th2-low asthma includes neutrophilic asthma and paucigranulocytic asthma. Although asthma is divided into Th2-high and Th2-low, both asthma phenotypes can occur simultaneously in some patients [21,22,28].

#### 1.2.2. Th1 Response

Th1 cells mainly secrete IL-2 and interferon-γ (IFN-γ) to protect the body from intracellular bacteria and viruses [29]. Epithelial damage promotes the maturation of Th1 cells and the secretion of Th1 cytokines, including tumor necrosis factor (TNF-α) and IFN-γ. TNF-α interacts with IL-17 cytokines to promote neutrophil recruitment. In addition, TNF-α enhances airway smooth muscle contraction [30,31]. It has been proven that IFN-γ secretion by Th1 cells is associated with the suppression of secretory leukocyte protease inhibitor, which is associated with airway hyperresponsiveness and pathological changes in the lungs [32,33].

#### 1.2.3. Th17 Response

Th17 cells produce both IL-17 and IL-22, which contribute to the development of asthma [6]. IL-17, through the activation of epithelial cells, fibroblasts, and smooth muscle cells, contributes to airway remodeling [34]. Additionally, IL-17A increases bronchial smooth muscle contraction [6,35]. However, it has been suggested that IL-17 is important for maintaining epithelial integrity; thus, IL-17 may play a protective role against asthma [36].

The asthma phenotype should be taken into account when choosing the appropriate treatment as the prognosis and response to drugs in eosinophilic and neutrophilic asthma are different.

### 1.3. Aim of the Study

Growing evidence suggests that EDN is more applicable than eosinophil blood count and ECP in evaluating disease severity [37]. VEGF may be an underappreciated pro-inflammatory factor contributing to asthma. Thus, the aim of this paper was to evaluate the biological function and role of EDN and VEGF in asthma pathogenesis. We also review the current knowledge about putative approaches of anti-VEGF and anti-eosinophil drugs in asthma therapy.

## 2. VEGF

### VEGF in Asthma Pathogenesis

Vascular endothelial growth factor (VEGF) belongs to a family of proteinaceous growth factors and is one of the most potent inducers of vasculogenesis and angiogenesis [38,39,40]. The cells involved in the synthesis and action of vascular endothelial growth factor are tumor cells, bronchial and lung epithelial cells, smooth muscle cells, vascular endothelial cells, macrophages, neutrophils, and Th2 lymphocytes. VEGF’s main targets are vascular endothelial cells, but its activity also includes monocytes, macrophages, embryonic stem cells, and neurons [41,42]. VEGF subfamily ligands’ most prominent structural feature is a cystine junction composed of three intertwined disulfide bridges [40,43].

**Figure 1:**
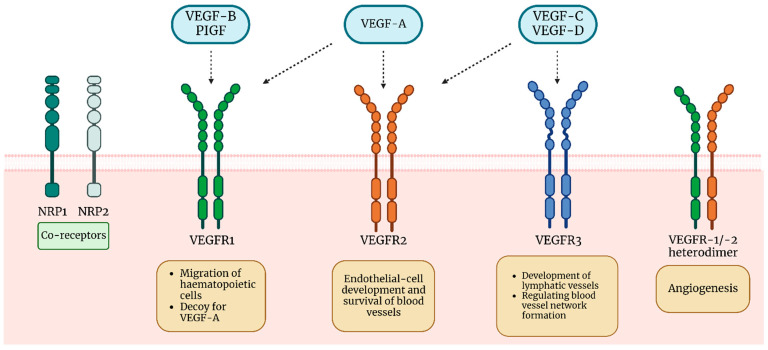
VEGF and corresponding receptors alongside their functions.

The VEGF subfamily consists of VEGF-A to VEGF-F and placental growth factor (PlGF) (Figure 1). However, we focus on VEGF-A due to its crucial role in angiogenesis, vasodilatation, the release of nitric oxide, and enhancing the chemotaxis of macrophages and granulocytes [39,44]. Hypoxia and inflammation stimulate hypoxia-inducible factors (HIFs), which can increase the activity of various proangiogenic factors through the HIF-1α pathway, as well as VEGF-A, becoming one of its primary regulators [45,46,47]. In addition to hypoxia-inducible factors (HIFs), there are other regulators of VEGF expression. One group with this function comprises growth factors, including epidermal growth factor, transforming growth factors (TGF) α and β, insulin-like growth factor-1, fibroblast growth factor, and platelet-derived growth factor (PDGF). During inflammation, interleukins (e.g., interleukin (IL)-1β or IL-6) are secreted, increasing VEGF expression in many cell types. VEGF modulation can also occur during oncogene mutations due to oncogenes (Ras) or tumor necrosis factor (TNF-α) [48]. The gene for VEGF-A is located on chromosome 6 and undergoes alternative splicing of exons to produce isoforms such as VEGF121, VEGF165, VEGF189, and VEGF206, of which VEGF165 is the most common isoform in tissues [39].

VEGF-B is involved in neovascularization occurring during embryonic development and in the progression of cancerous tumors. VEGF-C increases vascular permeability and the formation of lymphatic vessel networks [44]. VEGF-D is a tumor angiogenesis factor and promotes EC proliferation. It also modulates the abundance of lymphatic vessels in specific tissues during embryonic development. Understanding and confirming its other functions requires further research [44,49]. Placental growth factor (PLGF) may be involved in the growth and maintenance of pregnancy and wound healing [44]. Increased VEGF expression correlates with poorer clinical outcomes in many diseases; however, VEGF also has many possible therapeutic uses [39,50].

The VEGF receptors (VEGFRs) are members of the type III transmembrane tyrosine kinases (TKs) superfamily of receptors. For example, they are structurally related to PDGFR, IR, IGFR, or FGFR receptors [51,52]. VEGFRs consist of three subtypes: VEGFR-1 and VEGFR-2, both mainly found on vascular endothelial cells, and VEGFR-3, primarily located on lymphatic endothelial cells [52,53]. Structurally, VEGFRs are similar to each other. They have an extracellular domain consisting of seven Ig homology domains (D1–D7), a transmembrane domain, a juxtamembrane regulatory domain, and a tyrosine kinase domain [54,55].

The mechanism of VEGFR activation involves ligand-induced dimerization of the extracellular domain, followed by tyrosine autophosphorylation in the intracellular kinase domain to generate downstream signaling. The D2 domain serves mainly for ligand binding by VEGFR, with VEGFR-3 using the D1 domain for this purpose. The D3 serves to increase binding affinity. The D4-7 domains are involved in generating structural changes that are essential for receptor dimerization and activation [56,57]. Experiments on the receptor’s kinase domain have shown that domains 4–7 are not essential for signaling [58]. While VEGF-A binds with high affinity to VEGFR-1, it signals blood vessel development mainly through VEGFR-2 [54]. Conversely, VEGF-B and PIGF bind only to VEGFR-1 [59]. The growth factors primarily responsible for the development of lymphatic vessels are VEGF-C and VEGF-D, which signal especially through VEGFR-3 [60]. Studies conducted by Tammel et al. have shown that despite expression mainly on lymphatic vessels, VEGFR-3 is also involved in regulating blood vessel network formation, and its blockade may contribute to the efficacy of anti-angiogenic therapies [61].

An important function of VEGFR-1 is its role as a decoy for VEGF-A, serving as an endogenous anti-angiogenic factor by reducing its bioavailability to target cells. VEGFR-1, also produced in the placenta during pregnancy, binds to placental growth factor (PIGF) in the form of both VEGF/PIGF heterodimers and PIGF homodimers, which is important in, among other things, pre-eclampsia during pregnancy [62,63,64]. Numerous studies have also shown that under specific conditions, after stimulation by PIGF, VEGFR-1 can heterodimerize with VEGFR-2, transactivating it and positively regulating angiogenesis. In addition, PIGF can increase the expression of VEGF, further enhancing its positive effects on angiogenesis [65,66,67].

Important co-receptors for VEGF are neuropilins (NRPs). They are divided into NRP-1, expressed in arterial endothelial cells, and NRP-2, expressed in the endothelial cells of veins and lymphatic vessels. They are active protein receptors and can regulate neurogenesis and angiogenesis. The best-known binding is that of VEGF-A (specifically, VEGF-A165 or VEGF-A189) with NRP-1 in complex with VEGFR-1 and VEGFR-2. This results in forming a VEGF-A/VEGFR/NRP1 complex. Such a combination induces signal transduction downstream of the receptor, stimulating it and contributing to the activation of angiogenesis [48,68,69,70].

Research on the role of VEGF in the pathogenesis of bronchial asthma has shown that it can stimulate allergic inflammation in the bronchial tree and contribute to the remodeling processes. Numerous studies have also proven that asthmatic patients, including children, have significantly elevated levels of VEGF [71,72,73,74]. The study by Hoshino et al. demonstrated an increased number of vessels per unit area with no significant difference in mean vessel size in asthmatic patients compared to their number and size in non-asthmatic control subjects. Furthermore, they proved a correlation between the area of vessels and the amount of VEGF in the airways in asthmatic subjects [75]. The results of a study by Zhang et al. show that the highest levels of VEGF were observed in individuals during the acute phase of asthma, with lower levels in patients with stable asthma, and the lowest levels in the control group. In addition, by determining the sputum VEGF concentration in subjects with mild, moderate, or severe asthma, they proved a significant correlation between the VEGF-level increase and aggravation of the patient’s condition [76]. A different study confirmed the above results by showing elevated expression levels of both VEGF and its receptor VEGFR1 in the bronchial tissue of asthmatic patients. These correlated well with the extent of airway remodeling and airway hyperresponsiveness and were negatively correlated with lung function data. Patients showed submucosal gland hyperplasia, increased smooth muscle mass, increased reticular basement membrane thickness, subepithelial fibrosis, and neovascularization, compared with control subjects [77].

Mucins are responsible for mucus viscoelasticity, and their increased presence in the airways is due to mucus hypersecretion in asthma. MUC5AC is the main mucin glycoprotein overproduced in asthma. Studies have shown that VEGF increases MUC5AC mRNA expression in a dose- and time-dependent manner through the activation of RhoA kinases [78]. Asthma is connected with increased airway smooth muscle mass [79]. VEGF significantly affects matrix metalloproteinases MMP-1 and MMP-9 with a lesser impact on MMP-3. The endothelial growth factor increases their production, and studies suggest that this contributes to smooth muscle migration in angiogenesis [80].VEGF has also been confirmed to increase the expression of disintegrin and metalloproteinase (ADAM)-33, a factor that plays a significant role in the pathophysiology of asthma, as well as the proliferation of airway smooth muscle cells through activation of the VEGFR2/ERK1/2 pathway [81]. 

Genetic analyses of the airway smooth muscle cells in bronchial asthma patients have revealed epigenetic modifications of histones. The histone methylation pattern described during the study suggests that the VEGF promoter in asthmatic patients is more active and is also deprived of the repression mechanism present in the promoter of healthy patients. This mechanism involves the induction of histone H3K9 methylation by G9A recruitment, which results in reduced RNA pol II binding and reduced binding of Sp1, the VEGF promoter. This results in overexpression of the endothelial growth factor [82]. VEGF levels are also affected by claudin-1, whose elevated levels were observed in patients with asthma and a mouse model of asthma-like airway inflammation. Overexpression of claudin-1 resulted in increased proliferation of airway smooth muscle cells and significantly elevated levels of VEGF [83]. A study by de Paulis et al. demonstrated the possible influence of basophils on angiogenesis and inflammation through the synthesis and release of various VEGF isoforms from the cytoplasmic secretory granules of basophils. They also showed that some VEGF-A isoforms could function as chemotactors for basophils. Hence, this indicates that there is an essential autocrine loop that may participate in the development of asthma [84]. The effect of basophils on VEGF due to the previously mentioned hypoxia-inducible factor has also been demonstrated. The study showed that basophil activation by IgE results in the accumulation of the α subunit of hypoxia-inducible factor 1α (HIF-1α), which correlates with an increase in HIF-1α mRNA, as well as VEGF. Inhibition of HIF-1α expression in basophils showed that this protein is essential for the expression of vascular endothelial growth factor (VEGF) mRNA and, consequently, its release [85]. 

As mentioned above, interleukins also affect the concentration of VEGF in the body. For instance, interleukin 32 (IL-32) has been shown to affect VEGF levels. The study showed that silencing the previously mentioned interleukin in normal human bronchial epithelial cells increased VEGF secretion. Moreover, high levels of IL-32 correlated with better responses to treatment in patients with bronchial asthma. Accordingly, this implies that an adequate amount of IL-32 reduces endothelial growth factor production and angiogenesis [73]. Other studies have also proven that elevated IL-25 levels in asthma contribute to angiogenesis, at least in part by increasing VEGF/VEGF receptor expression in endothelial cells through the PI3K/Akt and Erk/MAPK pathways [86]. Interestingly, interleukin-5 has been shown to have anti-angiogenic potential. It has been shown to significantly inhibit vascular formation, including VEGF-induced endothelial cell proliferation, migration, and tube formation, acting through STAT5. Its blockade abolished the anti-angiogenic effect of IL-5 [87]. However, interleukin 33, whose elevated levels are induced by hypoxia, may partially increase the expression of HIF-1α and VEGF to initiate blood vessel remodeling [88]. Studies have confirmed the link between VEGF and the inflammatory response stimulated by Th2 lymphocytes. Furthermore, VEGF is a potent stimulator of inflammation and dysregulation while increasing the antigen sensitivity and inflammation of Th2 lymphocytes and increasing the number and activation of local dendritic cells. Studies have also revealed that VEGF production plays a significant role in forming Th2 cytokines and that epithelial cells and Th2 cells are potent producers of VEGF in the antigen-affected lung [89].

One paper summarized the relationships between micro-RNA, VEGF, and asthma. The meta-analysis results showed that some miRNAs have pro-angiogenic properties and can be stimulated by pro-angiogenic stimuli such as VEGF, among others. As an example, the expression of pro-angiogenic miR-130a or miR-132 increased under the influence of VEGF. Contrarily, miR-126 was described as having an anti-angiogenic effect, and its downregulation increased VEGF activity. The role and possible benefits of miRNAs in asthma are not yet well understood and require further and more thorough research [90].

It was also discovered that rhinovirus infection causes VEGF production in human airways, mainly by fibroblasts, which is further exacerbated in an atopic environment. The angiogenesis stimulated by rhinoviruses may cause the condition of patients with asthma to become worse [91,92].

Research conducted in China has revealed a correlation between VEGF polymorphisms and the prevalence of asthma in the Chinese Han population. The rs3025020 polymorphism of the VEGF gene was found to be related to asthma, and the frequency of the T allele in the asthma group was significantly higher than in the control group. Findings suggest that the VEGF rs3025020 polymorphism plays a significant role in cell proliferation and inappropriate VEGF-induced angiogenesis related to asthma [93]. Studies evaluated the association between VEGF polymorphisms and childhood asthma, lung function, and airway reactivity in two populations. In both of them, the rs833058 polymorphism was associated with asthma. It was also linked with increased airway reactivity among the subjects. Furthermore, it impacted the decrease in the ratio of forced expiratory volume in 1 s (FEV1)/forced vital capacity (FVC) over ~4.5 years of follow-up of the subjects [94]. In addition, it was shown that the presence of the rs3025028 polymorphism of the VEGF-A gene associated with airway function parameters measured in childhood correlates with the persistence of the effect after adulthood [95]. Moreover, subsequent studies of VEGF-A have shown a correlation between its gene polymorphisms and response to therapy with inhaled corticosteroids or leukotriene receptor antagonists (LTRAs). The AA genotype of the rs2146323 polymorphism correlated with a better response to corticosteroid therapy and a worse response to LTRA therapy. Furthermore, the rs833058 polymorphism was associated with a better response to episodic LTRA therapy [96].

## 3. EDN

### 3.1. EDN in Asthma Pathogenesis

**Figure 2:**
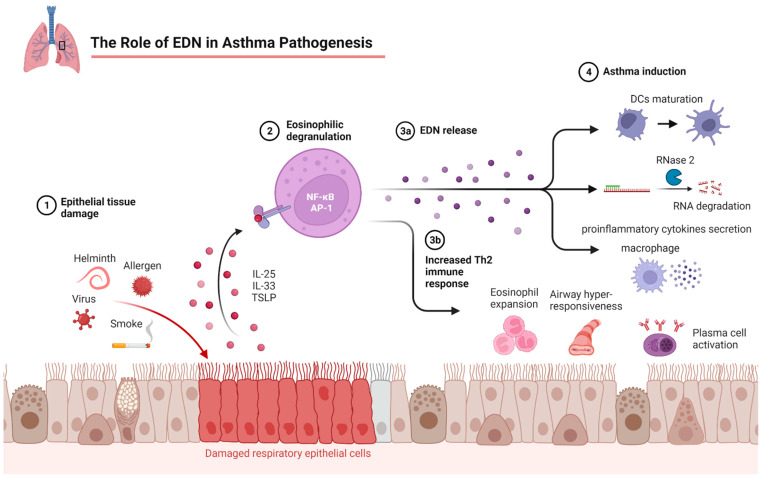
The role of EDN in asthma pathogenesis.

EDN (RNase2) belongs to the ribonuclease A superfamily [97]. EDN is a single-chain polypeptide with a molecular weight of 18.6 kDa. A comparison of the partial N-terminal amino acid sequences of EDN and ECP showed 67% identity and structural homology to pancreatic ribonuclease (RNase) [98,99]. EDN expression has been detected in the highest concentration in eosinophils. EDN expression was also found in monocytes, dendritic cells, basophils, and neutrophils [100,101]. In addition to its neurotoxic effects, EDN has been shown to have antiviral activity, particularly against the respiratory syncytial virus. As a result, it participates in the defense of the upper bronchial region against viral infection. Hence, it is suggested that EDN is involved in host defense against single-stranded RNA viruses [102,103,104,105]. In addition, EDN is associated with allergic airway inflammation [106]. In summary, EDN is a multifunctional mediator with cytotoxic, antiviral, and chemotactic effects on DCs [102] (Figure 2).

In patients with asthma, levels of type-2 instructional cytokines (IL-33 and IL-25) and effector cytokines (IL-4, IL-5, and IL-13) are elevated in the airway mucosa, associated with impaired antiviral immunity. In a study conducted in 2022, it was shown that exposure to eosinophils or eosinophil supernatants inhibited RV-induced IFN-α secretion by dendritic cells [107]. Eosinophil-secreted factors such as TGF-β and EDN suppressed the antiviral response by inhibiting CXCL10 and IFN-α production by dendritic cells (pDCs). The rhinovirus-stimulated pDCs secreted significantly less IFN-α and CXCL10 when co-cultured with eosinophils. Accordingly, this provided evidence that eosinophils attenuate the antiviral immunity of pDCs [108].

EDN is released from eosinophil granules after activation by cytokines (including IL-5, IL-11, and IL-33) and other proinflammatory mediators [109]. RNAase2 is selectively chemotactic for dendritic cells (DCs). Additionally, it induces the activation of mitogen-activated protein kinase p42/44 (MAPK) in DCs [102,110]. EDN can also induce the production of proinflammatory cytokines in monocytes/macrophages and the maturation of dendritic cells through Toll-like receptor 2 (TLR2) [111]. Studies suggest that eosinophil-derived neurotoxin also plays a role in allergic diseases [104]. Therefore, EDN promotes the allergic response by activating dendritic cells. EDN levels are closely related to Th2 inflammation [19]. Eosinophil-derived neurotoxins have been classified as alarmins, which are endogenous mediators rapidly released by cells of the host’s innate immune system in response to infection. Alarmins can activate antigen-presenting cells and enhance the immune system response [111,112,113].

### 3.2. EDN vs. Blood Eosinophil Counts in Asthma Control Status

Eosinophils play a central role in allergic diseases, so direct measurement of eosinophilic inflammation is essential for diagnosing, treating, and monitoring patients with asthma. Over recent years, specific markers have been identified and used to identify eosinophil activity and turnover. One of the markers has been EDN, which has been studied in a number of inflammatory diseases [114,115]. This neurotoxin, as a product of eosinophil degranulation, is attracting much attention as a new biomarker for diagnosing and monitoring asthma in children and adults [37].

In connection with the fact that EDN is secreted almost exclusively by eosinophils, this indicator may directly reflect exacerbations in eosinophilic inflammation [116].

Studies have suggested that airway inflammation associated with asthma exacerbation is characterized by an increase in eosinophils and an increase in eosinophil degranulation in the airways [37,117].

Measurement of eosinophils in sputum has been a widely used test to assess the status of patients with asthma. However, eosinophil levels may not always accurately represent the cellular state of the asthmatic airways [118]. It has been proven that the measurement of eosinophils in sputum or blood does not sufficiently correlate with the severity of airway inflammation in asthma, e.g., eosinophilia develops several weeks before an exacerbation of the disease [119,120]. It has been suggested that eosinophil secretory activity is equal to the product of the eosinophil concentration, and mediators, e.g., EDN and ECP, could be critical markers of disease severity [37,115,121].

Regardless of the asthma phenotype, when eosinophils are activated, they release EDN from their granules [37,100]. Recent studies have confirmed the efficacy of EDN as a marker of eosinophilic inflammation to monitor and treat asthma [116,122,123,124]. It was demonstrated that EDN could act as a biomarker reflecting lung function and as a biomarker positively correlated with asthma severity [114,125]. In a study by Kim et al., EDN levels were higher and lung function was decreased in patients with eosinophilic asthma exacerbations [125].

It has been shown that the EDN concentration test has high accuracy, and applicability on small-volume samples of specimens such as sputum, serum, and urine [126]. EDN measurement is an affordable test with practical application in the daily diagnosis, treatment, and monitoring of several eosinophil-related disorders, such as asthma and recurrent wheezing bronchiolitis caused by a respiratory syncytial virus (RSV) [114,127].

Serum levels of EDN were shown to be significantly different between patients with controlled and uncontrolled asthma [128]. A study by An et al. showed that the mean serum levels of EDN in the group with uncontrolled asthma were higher than those in the group with controlled asthma and healthy patients. Serum EDN levels were correlated with the total eosinophil count (TEC), but a receiver operating characteristic (ROC) curve analysis showed that serum EDN levels were significantly better at predicting uncontrolled asthma. This study demonstrates that EDN predicts asthma control better than blood eosinophil count [121]. In contrast, however, a study by Gon et al. did not indicate a significant correlation between blood eosinophil count or serum EDN with lung function and symptom scores in patients with asthma [129].

The diagnosis of allergic disease in young children is a difficult task. Examining airway function in children is particularly difficult because they cannot participate in various functional tests. It should be noted that poor asthma control leads to poor quality of life for children and their caregivers [130,131]. In the pediatric population, EDN may be a promising biomarker in distinguishing patients with persistent wheezing from those with wheezing caused by respiratory infections, and may be an aid in diagnosing school-age asthma [129,132].

It has been reported that children in the acute phase of asthma may have higher serum EDN levels than those in the stable phase, and in contrast to the TEC, serum EDN levels may be a predictor of asthma severity [124,133]. In addition, in children, EDN levels were significantly higher in the atopic asthma group than in the non-atopic asthma group or control patients [134].

## 4. Putative Anti-VEGF and Anti-Eosinophil Drugs in Asthma Therapy

### 4.1. Anti-VEGF

Given the above data, one can speculate that anti-VEGF treatment would benefit asthma therapy (Table 1). Glucocorticoids are the most effective medications currently available for the treatment of asthma. They act primarily as an anti-inflammatory and partially reduce the airway hyperresponsiveness characteristic of the condition [135,136]. For example, the use of budesonide in therapy significantly suppressed the spontaneous release of VEGF, and the production of VEGF increased by IL-4, IL-5, IL-13, TGF-β, or IL-1β [137]. Other studies have also confirmed that treatment with budesonide and formoterol reduces the expression of both VEGF and VEGFR1, which correlates with reduced airway remodeling in patients with asthma [77]. The previously mentioned rhinovirus is one of the stimulators of VEGF release. Budesonide has been shown to suppress the chemokines it induces, including VEGF [138].

Montelukast is a selective CysLT1 receptor antagonist used to treat asthma. One study investigated the effect of montelukast on the parameters of irritant-induced asthma induced by the inhalation of chlorine in mice. Montelukast inhibited this increase and effectively blocked the elevation of VEGF and IL-6 concentrations involved in inflammation via the CysLT1 receptor. However, the neutralization of IL-6, but not VEGF-A, attenuated chloride-induced neutrophilia and bronchial tree hyperresponsiveness in the lungs [139].

The study by Türkeli et al. may point to other treatment pathways for asthma. An experimental mouse model of asthma showed that anti-VEGF therapy effectively reduced growth factors and appeared to increase levels of the epithelial barrier proteins E-cadherin and β-catenin. For this reason, the use of anti-VEGF therapy in asthma seems to be a more effective treatment for epithelial barrier reconstruction and remodeling than, e.g., corticosteroid treatment and TNF-α inhibition, which were not effective in increasing E-cadherin and β-catenin levels [140]. 

Studies were also performed on the effect of licochalcone A on VEGF-induced respiratory smooth muscle cell proliferation. They showed that it inhibits the process, probably by blocking VEGFR2 and ERK1/2 activation and downregulating caveolin-1 [79].

Diosmetin has anti-inflammatory properties and may be another potential drug to treat asthma. Its administration resulted in a significant reduction not only in VEGF levels but also matrix metallopeptidase-9 and transforming growth factor-β1. Accordingly, this suggests a link between reduced airway remodeling and a decrease in these proteins [141].

Bevacizumab is an anti-VEGF monoclonal antibody that also shows potential in asthma treatment applications. Using a mouse model of house dust-mite (HDM)-induced asthma, it was found to reduce airway hyperresponsiveness and inflammation induced by HDM. In addition, it appeared to reduce the release of Th2 cytokines. The effect of bevacizumab administration may be due to the neutralization of VEGF-A and inhibition of VEGFR-2 activation [142]. The effective reduction of inflammation and reduced VEGF release is also supported by more recent studies, which suggest that inhibiting angiogenesis in rats with induced asthma not only suppresses the inflammatory process by blocking VEGF expression but also inhibits the development of new blood vessels and the progression of asthmatic attacks [143].

Another possible treatment option may be silver nanoparticles, which have been shown to reduce VEGF signaling through the PI3K/HIF-1α/VEG pathway, EGFR levels, and MUC5AC expression, while having minimal toxicity [144]. Studies have also shown that silver nanoparticles can have an anti-angiogenic effect. In this case, the effect was confirmed by inducing the death of primary bovine retinal endothelial cells, even in the presence of VEGF, by up to 50%. In the same model, it was also proven that silver nanoparticles inhibit VEGF-induced cell proliferation and migration. In the VEGF-treated material, increased endothelial cell migration and complete wound closure were observed no later than 24 h after treatment, while in the silver-nanoparticle-treated samples, a vast area of the wound remained exposed [145].

The effect of pigment epithelium-derived factor (PEDF) on airway remodeling in chronic allergic asthma was also studied. It was found that PEDF has an inhibitory effect on eosinophil-induced airway inflammation, airway hyperreactivity, and airway remodeling. In addition, mice experienced a significant inhibitory effect on ovalbumin-stimulated VEGF production. PEDF also inhibited VEGF release from IL-1β-stimulated BEAS-2B cells [146]. 

Administration of insulin-like growth factor (IGF) binding protein, specifically IGFBP-3, reduced HIF-1α/HIF-2α signaling, which mediates VEGF expression, and IGF-I production, contributing to reduced VEGF expression, airway inflammation, and bronchial hyperreactivity [147].

The next potential drug is epigallocatechin gallate (EGCG). EGCG is the active catechin found in green tea. In a paper by Yang et al., the protective effect of EGCG against HDM-induced asthma was studied in mice. The results showed that it alleviates tissue damage, inflammation, mucus production, and collagen deposition, and reduces M2 macrophage infiltration. The tested compound alleviates asthma symptoms in mice by suppressing HIF-1α/VEGFA-mediated M2 macrophage skewing. Hence, this confirms that restoration of both HIF-1α and VEGFA significantly blocked the protective functions of EGCG [148]. EGCG has also been shown to reduce lung injury and airway remodeling in asthmatic rats exposed to PM2.5 and to have a protective effect on the lungs. This mechanism may be associated with regulation of the HMGB1/RAGE signaling pathway [149].

There have also been studies on mice that have shown a beneficial effect of prolonged azithromycin (AZT) administration on airway remodeling. This is achieved through the PI3K/Akt/mTOR/HIF-1α/VEGF pathway. Reduced airway reactivity and fewer lesions were observed, as confirmed by significantly reduced HIF-1α and VEGF levels [150]. Other studies have also confirmed the beneficial effects of AZT, revealing its anti-angiogenic effect through the p38(MAPK) pathway caused by fibroblast growth factor stimulation [151]. Long-term low-dose azithromycin therapy has been proven to be beneficial in patients with different endotypes of severe asthma. A substantial reduction in the number of asthma exacerbations during therapy and an improvement in their condition were observed. AZT is less toxic than maintenance inhaled corticosteroids, so it could be used before them in severe non-eosinophilic asthma [152]. The effect of AZT on poorly controlled asthma in children has also been studied. The use of AZT in children resulted in improved asthma control and fewer exacerbations without significant side effects. The beneficial effect of AZT was similar in children with both eosinophilic and non-eosinophilic asthma [153].

Other studies have shown that vitamin D inhibits VEGF-induced respiratory smooth muscle cell proliferation by suppressing VEGFR2 and ERK1/2 activation and downregulation of ADAM33, which may be crucial in developing therapies for diseases such as asthma [154].

Studies of the shark-cartilage-derived anti-angiogenic drug Neovastat (AE-941) in the treatment of asthma have also been conducted. Reports have shown its ability to suppress the activity of HIF-2α, one of the hypoxia-induced factors that increase VEGF levels. However, the efficacy and feasibility of Neovastat need to be confirmed by more research [155]. 

The effect of endostatin/Fc on ovalbumin-induced cellular immunization was examined in a mouse model of asthma. It was shown to inhibit airway hyperreactivity, allergic pulmonary inflammation, ovalbumin-specific IgE production, and lung inflammation mediators. However, data from the study show only partial inhibition of asthma features by the VEGF receptor blockade, indicating that it is not only the result of VEGF antagonism signaling but may also act through other mechanisms [156].

Recently, the effect of melatonin on airway remodeling in asthma has been studied. The results indicate that melatonin significantly reduces airway hyperreactivity, inflammation, and remodeling in a house dust-mite model. Melatonin was also found to significantly inhibit airway smooth muscle cell proliferation, VEGF synthesis, and PDGF-induced cell migration, which may depend on STAT3 signaling. These studies point to the future possibility of using melatonin to treat asthma [157].

**Table 1:** Summary of drugs with anti-VEGF activity in asthma.

Drug	Mechanism	Response to Treatment	Adverse Events/Toxicity	Dose	Dose Dependence	Remarks	References
Budesonide	↓VEGF ↓VEGFR-1	↑FEV(1) ↓airway hyperresponsiveness	well-tolerated	100 μg of budesonide + 12 μg of formoterol twice daily or 400 μg of budesonide plus placebo twice daily	at follow-up, clinical and functional outcome measures were better in the group treated with low-dose budesonide + formoterol, compared to the higher dose of budesonide	drug in use	[77,137,138,158]
Licochalcone A	↓VEGFR-2 ↓ERK1/2 ↓caveolin-1	ND	ND	10, 20 and 30 μM of licochalcone A per 50 ng/mL of VEGF	the higher doses had a better effect	more studies on humans need to be conducted	[79]
Silver nanoparticles	↓VEGF ↓EGFR ↓MUC5AC	ND	research on mice showed increased pro-inflammatory T17 responses	silver NPs were administered for 48 h with different doses (10, 20, 50, 100, 200, or 500 μM)	elevated levels of HIF-1α, VEGF, and PI3K were significantly reduced in a dose-dependent manner	antiangiogenic effect, potential toxicity depending on genotype and phenotype	[144,145,159]
Bevacizumab	↓VEGF-A ↓VEGFR-2	ND	reversible bronchospasm	5 mg/kg body weight	ND	further investigation of the signaling pathway involved in cytokine release and immunoregulation is required	[142,160]
Azithromycin	↓HIF-1α ↓VEGF	↓airway reactivity and lesions ↓exacerbations and improved quality of life when treated with oral azithromycin in adults with persistent symptomatic asthma ↓respiratory infections	well-tolerated, increased risk of cardiac torsades des pointes in patients at risk (prolongation of QT interval) and diarrhea	500 mg three times per week	long-term, low-dose azithromycin reduces asthma exacerbations	the long-term effects on community microbial resistance require further studies	[150,161,162]
Diosmetin	↓VEGF ↓TGF-β1	↓airway remodeling ↓fibrogenesis	ND	0.5 and 0.1 mg/kg	the high dose of diosmetin (0.5 mg/kg) significantly decreased the numbers of total cells, eosinophils, and neutrophils, whereas the low dose of diosmetin (0.1 mg/kg) had only a slight effect	antiproliferative effect on airway smooth muscle cells, antioxidant activity; more research needs to be conducted	[141,163,164]
Vitamin D	↓VEGF-induced respiratory smooth muscle cell proliferation ↓disintegrin and metalloproteinase (ADAM33)	add-on therapy enhances corticosteroid responsiveness	in case of an overdose	20, 50, 100 nM	inhibited the release of VEGF from ASM cells stimulated with TGF-β1 in a dose-dependent manner	supplementation did not prevent severe asthma exacerbations in children; more research needs to be conducted	[154,165,166]
Epigallocatechin gallate	↓HIF-1α/VEGFA-mediated M2 macrophage skewing in mice ↓TGF-β1	ND	ND	10 mg/kg and 50 mg/kg	results indicate that EGCG, especially high-dose EGCG, could alleviate lung injury caused by PM2.5 exposure to asthma	exhibits antiviral, antibacterial, antioxidative, anticancer and chemopreventive activities; more research needs to be conducted	[148,149,167,168]
IGFBP-3	↓HIF-1α/HIF-2α ↓IGF-I ↓VEGF ↓NF-κB signaling pathway	↓airway inflammation ↓airway hyperresponsiveness	ND	10 μg/kg or 50 μg/kg body weight per day	higher dose had a better effect	more research needs to be conducted	[147,169]
Neovastat (AE-941)	↓HIF-2α ↓VEGF	ND	ND	5 mg/kg	ND	anticancer, antimetastatic antiangiogenic activity; more research needs to be conducted	[155,170]
Endostatin/Fc	↓VEGFR-1,-2,-3	↓airway inflammation ↓airway hyperresponsiveness	ND	20 mg/kg twice a day	ND	antiangiogenic activity, in use in other diseases, possible use in the treatment of asthma requires further study	[156]
Montelukast	↓VEGF ↓IL-6 ↓IL-4 ↓IL-13 ↓eotaxin	↓airway inflammation ↓airway hyperresponsiveness ↓mucus production ↓pulmonary fibrosis	agitation, anxiety, depression, sleep disturbance, hallucinations, suicidal thinking and suicidality, tremor, dizziness, drowsiness, neuropathies, seizures, anaphylaxis, and eosinophilic infiltration	2 mg, 10 mg, 50 mg	10 and 50 mg doses were associated with similar improvement, while the 2 mg dose was less effective and not significantly different from the placebo	IL-6 appears to have a greater effect in blocking irritant-induced asthma	[139,171,172]
PEDF	↓VEGF	↓airway inflammation ↓airway hyperresponsiveness ↓airway remodeling	ND	50 or 100 µg/kg body weight	PEDF inhibited the release of VEGF from BEAS-2B cells stimulated with IL-1β in a dose-dependent manner	antiangiogenic, anticancer, and pro-differentiation factor; more research needs to be conducted	[146,173]
Melatonin	↓VEGF ↓TRPV1 channel ↓MUC5AC ↓MAPK signaling	↓airway inflammation ↓airway hyperresponsiveness ↓airway remodeling	ND	15 mg/kg body weight	ND	could antagonize ozone-exacerbated asthma, antioxidative, antiproliferative factor; more research needs to be conducted	[157,174,175]

ND—no data; TRPV1—transient receptor potential vanilloid 1; ERK—extracellular signal-regulated kinase; FEV(1)—forced expiratory volume in one second; MAPK—mitogen-activated protein kinase; MUC5AC—mucin 5AC.

### 4.2. Anti-Eosinophil Drugs

Eosinophils are essential effector cells in type 2 inflammation with IL-4, IL-5, and IL-13 as pivotal cytokines. Over the past decade, several specific monoclonal antibodies and small-molecule drugs have been introduced to target those proteins. Among them are anti-IL-5 or anti-IL-5 receptor α (mepolizumab, reslizumab, and benralizumab), anti-IL-13 (lebrikizumab and tralokinumab), anti-IL-4 receptor α (dupilumab), anti-IgE (omalizumab), anti-thymic stromal lymphopoietin (tezepelumab), and small-molecule drugs such as prostaglandin D2 blockers (fevipiprant and timapiprant) [176] (Table 2). Therapy involving the neutralization of those cytokines reduces the frequency of asthma exacerbations (AEs) [108,177]. Airway eosinophilia often predicts responses to therapies using anti-IL-5 monoclonal antibodies [178]. EAACI established a blood eosinophil cut-point of ≥150/μL for a conditional recommendation to guide anti-IL-5 initiation in adult patients with severe asthma. Specific eosinophil (≥260/μL) and FeNO (≥19.5 ppb) cut-offs are suggested to identify adolescents or adults with the highest likelihood for response to anti-IgE therapy [13].

EDN levels have been shown to decrease after treatment with budesonide and benralizumab [179]. Benralizumab is a monoclonal antibody directed against interleukin-5Rα, which selectively reduces the number of eosinophils through increased antibody-dependent cellular cytotoxicity. The drug reduces the frequency of severe asthma exacerbations and lowers the daily dose of corticosteroids [180]. In a study, benralizumab used in patients with asthma was shown to reduce blood eosinophil counts and serum EDN and ECP from baseline values [181].

Mepolizumab is also a monoclonal antibody directed against IL-5. It has been shown to reduce the frequency of AE by about half in participants with severe eosinophilia and improve patients’ quality of life [182]. A study by Gon et al. showed a significant correlation between the reduction in serum EDN from baseline and improved lung function after omalizumab treatment [129].

Another study of 15 patients with severe eosinophilic asthma showed that after 6-month treatment with reslizumab, total eosinophil count (TEC) and EDN decreased with a significant increase in FEV(1). Despite the small study group, these findings suggest that EDN is a valuable biomarker for monitoring anti-IL-5 treatment [178].

**Table 2:** Summary of drugs with anti-eosinophil activity in asthma.

Drug	Mechanism	Response to Treatment	Adverse Events/Toxicity Reported	Remarks	Dose	Dose Dependence	References
Budesonide	↓IL-5 ↓TEC ↓IFN-γ ↓EDN	↑FEV(1) ↓airway hyperresponsiveness	well-tolerated	effective only in non-smokers	400 μg/d, 800 μg/d	400 μg/d reduced the number of IL-5-responsive progenitor cells in the bone marrow at baseline; 800 μg/d reduced circulating eosinophils and serum levels of IL-5, as well as the recruitment of eosinophils to the airway after the allergen challenge	[183,184,185]
Benralizumab	↓IL-5 ↓TEC ↓EDN ↓ECP	↑FEV(1) ↓AER	well tolerated, injection-site reaction	recommended for 12+ years old children and adults with severe eosinophilic asthma	25, 30, 100, or 200 mg every 4 weeks	blood eosinophils and sera EDN concentrations were significantly decreased after benralizuma treatment (25, 100, or 200 mg) relative to baseline and were dosage-independent (*p* < 0.05)	[13,186,187,188,189,190]
Mepolizumab	↓IL-5 ↓EDN	↑FEV(1), ↓AER improved ACQ	nasopharyngitis, headache, injection-site reaction	recommended for 12+ years old children and adults with severe eosinophilic asthma, men or people having BMI ≥30 had a lesser response	75 mg, 100, 250 mg, 750 mg every 4 weeks	dose of 750 mg achieved the greatest reduction, dose-related reduction in sputum eosinophils	[13,191,192,193,194,195]
Reslizumab	↓IL-5 ↓EDN ↓TEC	↑FEV(1), ↓AER	anaphylaxis	recommended for adults with severe eosinophilic asthma	100 mg, 3 mg/kg intravenously every 4 weeks	no significant difference	[13,177,190,191,196,197]
Dupilumab	↓IL-4 ↓IL-13 ↓serum IgE, ↓plasma eotaxin-3	↑FEV(1), ↓AER ↓FeNO,	injection-site reaction, nasopharyngitis, injection-site erythema, bronchitis, hypereosinophilia	recommended for 12+ years old children and adults with severe eosinophilic asthma, especially effective in patients with high levels of blood eosinophils and FENO	100 mg for those weighing ≤30 kg, 200 mg every 2 weeks, 300 mg every 2 weeks	no significant difference	[13,190,198,199,200,201]
Lebrikizumab	↓IL-13	↑FEV(1) ↓AER	injection-site reaction	did not always show a significant reduction in asthma exacerbations in biomarker-high patients	37.5 mg, 125 mg, 250 mg	37.5 mg (81% reduction of exacerbation rate), 125 mg (77% reduction), 250 mg (no significant reduction)	[202,203,204,205]
Tralokinumab	↓IL-13	↑FEV(1) ↑FVC ↓AER	well-tolerated	promising results in atopic dermatitis	150 mg, 300 mg, 600 mg subcutaneously every 2 weeks	only tralokinumab 600 mg improved FEV1 significantly	[206,207,208]
Omalizumab	↓EDN ↓serum IgE	↑FEV(1), ↑FVC ↑PEF ↓AER	anaphylaxis	recommended for adults with severe eosinophilic asthma	75–375 mg subcutaneously every two or four weeks	the basis for dose and frequency is calculated considering patient weight and pretreatment total IgE serum levels; a minimum dose of 0.008 mg/kg of body weight per IgE (IU/mL) every 2 weeks or 0.016 mg/kg per IgE (IU/mL) every 4 weeks	[13,129,209,210,211]
Tezepelumab	↓TSLP ↓IL-5 ↓IL-13 ↓IgE	↑FEV(1) ↓AER ↓FeNO	nasopharyngitis, upper respiratory tract infection, headache, injection-site reaction	add-on maintenance treatment for 12+ years old children and adults	70 mg, 210 mg, 280 mg every 4 weeks	annualized asthma exacerbation rates at week 52 were 0.27 (70 mg), 0.20 (210 mg), and 0.23 (280 mg), compared with 0.72 in the placebo group; 210 mg dose reduced exacerbation rates by 64–82%	[206,212,213]
Fevipiprant	↓PGD2	↑FEV(1) ↓AER improved ACQ	well-tolerated	not recommended for routine use, further studies are needed	150 mg/d, 450 mg/d	RR of asthma exacerbation: 0.77 (0.61–0.97) for 450 mg compared with placebo; 0.86 (0.69–1.08) for 150 mg dose	[214]

AER—annualized exacerbation rate; FEV(1)—forced expiratory volume in one second; ACQ—Asthma Control Questionnaire; TSLP—human thymic stromal lymphopoietin receptor; TEC—total eosinophil count; PGD2—prostaglandin D2; FeNO—fractional exhaled nitric oxide.

## 5. Discussion

Elevated VEGF levels have been found in countless diseases, e.g., neoplasms, osteoarthritis, neovascular age-related macular degeneration, ischemic stroke, and post-hemorrhagic hydrocephalus [215,216,217,218,219,220,221]. Regarding lung diseases, patients with pulmonary hypertension, acute respiratory distress syndrome (ARDS), chronic obstructive pulmonary disease (COPD), lung cancer, and asthma have altered VEGF levels [41,222]. Interestingly, whereas asthma patients present with increased VEGF levels, Th2 and predominant eosinophilic phenotype, and hypervascularity, COPD patients are found to have decreased VEGF levels, prevailing Th1 and neutrophilic response, and loss of alveolar septal capillaries with emphysema [41,223].

Anti-VEGF therapy has been utilized in the treatment of neovascular age-related macular degeneration, diabetic retinopathy, polypoidal choroidal vasculopathy, retinopathy of prematurity, neovascular glaucoma, hepatocellular carcinoma, and prostate cancer [224,225,226,227,228,229,230]. Accordingly, anti-VEGF drugs are mostly used in the treatment of eye disorders and neoplasms. Regarding asthma, further studies and clinical trials are needed to evaluate the efficacy of add-on therapy with lichochalcone A, bevacizumab, diosmetin, epigallocatechin gallate, IGFBP-3, Neovastat, endostatin, PEDF, and melatonin. 

Biologic anti-eosinophil drugs in asthma have recently aroused much interest. The Food and Drug Administration (FDA) has approved omalizumab, mepolizumab, reslizumab, benralizumab, dupilumab, and tezepelumab for asthma treatment. These monoclonal antibodies have been shown to improve asthma control, decrease asthma exacerbation rates, and reduce glucocorticoid dependence [231,232]. Due to the heterogeneity of the asthma clinical picture and treatment responses, identifying the appropriate patients for these drugs is crucial.

We believe that the drugs listed herein constitute a promising strategy for the treatment of severe eosinophilic asthma. However, this therapeutic approach has limitations. To date, only a few human studies and clinical trials on several reviewed drugs have been conducted in asthma. Further studies are required to corroborate their efficacy and long-term safety. Another limitation is the lack of data regarding sex, age, BMI, comorbidities, smoking status, allergic history, and disease severity in some reviewed papers. Moreover, asthma and COPD overlap syndrome accounts for 15–25% of obstructive pulmonary diseases [233,234,235,236]. We conclude that the proposed management would not be successful in those patients. 

## 6. Conclusions

Asthma is a multifactorial disease with a pathogenesis involving a multitude of cytokines, chemokines, and growth factors. Approximately 65% of patients have poorly controlled asthma. Thus, there is a need to elaborate novel therapeutic and diagnostic strategies. VEGF and EDN levels are elevated in asthmatics and have a significant influence on modulating inflammatory processes. Several currently used therapeutics, e.g., budesonide and montelukast, have anti-VEGF properties. Among others with such activity are lichochalcone A, bevacizumab, diosmetin, epigallocatechin gallate, IGFBP-3, Neovastat, endostatin, PEDF, and melatonin. Azithromycin (AZT) at 500 mg for 48 weeks (3 times/week) reduces exacerbation rates and may be considered in adults with persistent symptomatic asthma. However, the long-term effects of AZT on community microbial resistance require further investigation. Vitamin D supplementation may enhance corticosteroid responsiveness. EDN is recommended to monitor asthma control status and anti-eosinophil drug therapy in children and adults. More research is needed to evaluate the efficacy of add-on therapy with listed anti-VEGF and anti-eosinophil drugs.

## Data Availability

Data sharing is not applicable as no datasets were generated or analysed during the current study.

## References

[1] Vos T., Abajobir A.A., Abbafati C., Abbas K.M., Abate K.H., Abd-Allah F., Abdulle A.M., Abebo T.A., Abera S.F., Aboyans V. (2017). Global, Regional, and National Incidence, Prevalence, and Years Lived with Disability for 328 Diseases and Injuries for 195 Countries, 1990-2016: A Systematic Analysis for the Global Burden of Disease Study 2016. Lancet.

[2] Song P., Adeloye D., Salim H., Dos Santos J.P., Campbell H., Sheikh A., Rudan I. (2022). Global, Regional, and National Prevalence of Asthma in 2019: A Systematic Analysis and Modelling Study. J. Glob. Health.

[3] Abbafati C., Abbas K.M., Abbasi-Kangevari M., Abd-Allah F., Abdelalim A., Abdollahi M., Abdollahpour I., Abegaz K.H., Abolhassani H., Aboyans V. (2020). Global Burden of 369 Diseases and Injuries in 204 Countries and Territories, 1990-2019: A Systematic Analysis for the Global Burden of Disease Study 2019. Lancet.

[4] Lee Y.J., Fujisawa T., Kim C.K. (2019). Biomarkers for Recurrent Wheezing and Asthma in Preschool Children. Allergy Asthma Immunol. Res..

[5] Wu T.D., Brigham E.P., McCormack M.C. (2019). Asthma in the Primary Care Setting. Med. Clin. N. Am..

[6] Gans M.D., Gavrilova T. (2020). Understanding the Immunology of Asthma: Pathophysiology, Biomarkers, and Treatments for Asthma Endotypes. Paediatr. Respir. Rev..

[7] Ntontsi P., Photiades A., Zervas E., Xanthou G., Samitas K. (2021). Genetics and Epigenetics in Asthma. Int. J. Mol. Sci..

[8] Maslan J., Mims J.W. (2014). What Is Asthma? Pathophysiology, Demographics, and Health Care Costs. Otolaryngol. Clin. N. Am..

[9] Habib N., Pasha M.A., Tang D.D. (2022). Current Understanding of Asthma Pathogenesis and Biomarkers. Cells.

[10] Popović-Grle S., Štajduhar A., Lampalo M., Rnjak D. (2021). Biomarkers in Different Asthma Phenotypes. Genes.

[11] Schoettler N., Strek M.E. (2020). Recent Advances in Severe Asthma: From Phenotypes to Personalized Medicine. Chest.

[12] Jeong J., Lee H.K. (2021). The Role of CD4+ T Cells and Microbiota in the Pathogenesis of Asthma. Int. J. Mol. Sci..

[13] Agache I., Akdis C.A., Akdis M., Canonica G.W., Casale T., Chivato T., Corren J., Chu D.K., Del Giacco S., Eiwegger T. (2021). EAACI Biologicals Guidelines-Recommendations for Severe Asthma. Allergy.

[14] Carr T.F., Zeki A.A., Kraft M. (2018). Eosinophilic and Noneosinophilic Asthma. Am. J. Respir. Crit. Care Med..

[15] Rothenberg M.E., Hogan S.P. (2006). The Eosinophil. Annu. Rev. Immunol..

[16] Jacobsen E.A., Ochkur S.I., Lee N.A., Lee J.J. (2007). Eosinophils and Asthma. Curr. Allergy Asthma Rep..

[17] Wegmann M. (2011). Targeting Eosinophil Biology in Asthma Therapy. Am. J. Respir. Cell Mol. Biol..

[18] Karawajczyk M., Peterson C.G.B., Venge P., Garcia R.C. (2013). An Extragranular Compartment of Blood Eosinophils Contains Eosinophil Protein X/Eosinophil-Derived Neurotoxin (EPX/EDN). Inflammation.

[19] Tsuda T., Maeda Y., Nishide M., Koyama S., Hayama Y., Nojima S., Takamatsu H., Okuzaki D., Kinehara Y., Kato Y. (2019). Eosinophil-Derived Neurotoxin Enhances Airway Remodeling in Eosinophilic Chronic Rhinosinusitis and Correlates with Disease Severity. Int. Immunol..

[20] Mims J.W. (2015). Asthma: Definitions and Pathophysiology. Int. Forum Allergy Rhinol..

[21] Woodruff P.G., Modrek B., Choy D.F., Jia G., Abbas A.R., Ellwanger A., Arron J.R., Koth L.L., Fahy J.V. (2009). T-Helper Type 2-Driven Inflammation Defines Major Subphenotypes of Asthma. Am. J. Respir. Crit. Care Med..

[22] Fahy J.V. (2015). Type 2 Inflammation in Asthma—Present in Most, Absent in Many. Nat. Rev. Immunol..

[23] Ramakrishnan R.K., Al Heialy S., Hamid Q. (2019). Role of IL-17 in Asthma Pathogenesis and Its Implications for the Clinic. Expert Rev. Respir. Med..

[24] Lambrecht B.N., Hammad H., Fahy J.V. (2019). The Cytokines of Asthma. Immunity.

[25] Yamauchi K., Ogasawara M. (2019). The Role of Histamine in the Pathophysiology of Asthma and the Clinical Efficacy of Antihistamines in Asthma Therapy. Int. J. Mol. Sci..

[26] Varricchi G., Bagnasco D., Borriello F., Heffler E., Canonica G.W. (2016). Interleukin-5 Pathway Inhibition in the Treatment of Eosinophilic Respiratory Disorders: Evidence and Unmet Needs. Curr. Opin. Allergy Clin. Immunol..

[27] Conde E., Bertrand R., Balbino B., Bonnefoy J., Stackowicz J., Caillot N., Colaone F., Hamdi S., Houmadi R., Loste A. (2021). Dual Vaccination against IL-4 and IL-13 Protects against Chronic Allergic Asthma in Mice. Nat. Commun..

[28] McGrath K.W., Icitovic N., Boushey H.A., Lazarus S.C., Sutherland E.R., Chinchilli V.M., Fahy J.V. (2012). A Large Subgroup of Mild-to-Moderate Asthma Is Persistently Noneosinophilic. Am. J. Respir. Crit. Care Med..

[29] Knochelmann H.M., Dwyer C.J., Bailey S.R., Amaya S.M., Elston D.M., Mazza-McCrann J.M., Paulos C.M. (2018). When Worlds Collide: Th17 and Treg Cells in Cancer and Autoimmunity. Cell. Mol. Immunol..

[30] Sieck G.C., Dogan M., Young-Soo H., Osorio Valencia S., Delmotte P. (2019). Mechanisms Underlying TNFα-Induced Enhancement of Force Generation in Airway Smooth Muscle. Physiol. Rep..

[31] Niessen N.M., Gibson P.G., Baines K.J., Barker D., Yang I.A., Upham J.W., Reynolds P.N., Hodge S., James A.L., Jenkins C. (2021). Sputum TNF Markers Are Increased in Neutrophilic and Severe Asthma and Are Reduced by Azithromycin Treatment. Allergy.

[32] Raundhal M., Morse C., Khare A., Oriss T.B., Milosevic J., Trudeau J., Huff R., Pilewski J., Holguin F., Kolls J. (2015). High IFN-γ and Low SLPI Mark Severe Asthma in Mice and Humans. J. Clin. Investig..

[33] Hudey S.N., Ledford D.K., Cardet J.C. (2020). Mechanisms of Non-Type 2 Asthma. Curr. Opin. Immunol..

[34] Chang Y., Al-Alwan L., Risse P.A., Roussel L., Rousseau S., Halayko A.J., Martin J.G., Hamid Q., Eidelman D.H. (2011). TH17 Cytokines Induce Human Airway Smooth Muscle Cell Migration. J. Allergy Clin. Immunol..

[35] Kudo M., Melton A.C., Chen C., Engler M.B., Huang K.E., Ren X., Wang Y., Bernstein X., Li J.T., Atabai K. (2012). IL-17A Produced by Aβ T Cells Drives Airway Hyper-Responsiveness in Mice and Enhances Mouse and Human Airway Smooth Muscle Contraction. Nat. Med..

[36] Hynes G.M., Hinks T.S.C. (2020). The Role of Interleukin-17 in Asthma: A Protective Response?. ERJ Open Res..

[37] Kim C.K. (2013). Eosinophil-Derived Neurotoxin: A Novel Biomarker for Diagnosis and Monitoring of Asthma. Korean J. Pediatr..

[38] Lee C.G., Ma B., Takyar S., Ahangari F., DelaCruz C., He C.H., Elias J.A. (2011). Studies of Vascular Endothelial Growth Factor in Asthma and Chronic Obstructive Pulmonary Disease. Proc. Am. Thorac. Soc..

[39] Apte R.S., Chen D.S., Ferrara N. (2019). VEGF in Signaling and Disease: Beyond Discovery and Development. Cell.

[40] Yamazaki Y., Morita T. (2006). Molecular and Functional Diversity of Vascular Endothelial Growth Factors. Mol. Divers..

[41] Voelkel N.F., Vandivier R.W., Tuder R.M. (2006). Vascular Endothelial Growth Factor in the Lung. Am. J. Physiol. Lung Cell. Mol. Physiol..

[42] Tuder R.M., Yun J.H. (2008). Vascular Endothelial Growth Factor of the Lung: Friend or Foe. Curr. Opin. Pharmacol..

[43] Vitt U.A., Hsu S.Y., Hsueh A.J.W. (2001). Evolution and Classification of Cystine Knot-Containing Hormones and Related Extracellular Signaling Molecules. Mol. Endocrinol..

[44] Iyer S., Acharya K.R. (2011). Tying the Knot: The Cystine Signature and Molecular-Recognition Processes of the Vascular Endothelial Growth Factor Family of Angiogenic Cytokines. FEBS J..

[45] Zhou Y., Zhu X., Cui H., Shi J., Yuan G., Shi S., Hu Y. (2021). The Role of the VEGF Family in Coronary Heart Disease. Front. Cardiovasc. Med..

[46] Mayer G. (2011). Capillary Rarefaction, Hypoxia, VEGF and Angiogenesis in Chronic Renal Disease. Nephrol. Dial. Transpl..

[47] Long H.Q., Li G.S., Cheng X., Xu J.H., Li F.B. (2015). Role of Hypoxia-Induced VEGF in Blood-Spinal Cord Barrier Disruption in Chronic Spinal Cord Injury. Chin. J. Traumatol..

[48] Uemura A., Fruttiger M., D’Amore P.A., De Falco S., Joussen A.M., Sennlaub F., Brunck L.R., Johnson K.T., Lambrou G.N., Rittenhouse K.D. (2021). VEGFR1 Signaling in Retinal Angiogenesis and Microinflammation. Prog. Retin. Eye Res..

[49] Baldwin M.E., Halford M.M., Roufail S., Williams R.A., Hibbs M.L., Grail D., Kubo H., Stacker S.A., Achen M.G. (2005). Vascular Endothelial Growth Factor D Is Dispensable for Development of the Lymphatic System. Mol. Cell. Biol..

[50] Ferrara N. (1999). Role of Vascular Endothelial Growth Factor in the Regulation of Angiogenesis. Kidney Int..

[51] Elebiyo T.C., Rotimi D., Evbuomwan I.O., Maimako R.F., Iyobhebhe M., Ojo O.A., Oluba O.M., Adeyemi O.S. (2022). Reassessing Vascular Endothelial Growth Factor (VEGF) in Anti-Angiogenic Cancer Therapy. Cancer Treat. Res. Commun..

[52] Li E., Hristova K. (2010). Receptor Tyrosine Kinase Transmembrane Domains: Function, Dimer Structure and Dimerization Energetics. Cell Adh. Migr..

[53] Takahashi S. (2011). Vascular Endothelial Growth Factor (VEGF), VEGF Receptors and Their Inhibitors for Antiangiogenic Tumor Therapy. Biol. Pharm. Bull..

[54] Secker G.A., Harvey N.L. (2021). Regulation of VEGFR Signalling in Lymphatic Vascular Development and Disease: An Update. Int. J. Mol. Sci..

[55] Cébe-Suarez S., Zehnder-Fjällman A., Ballmer-Hofer K. (2006). The Role of VEGF Receptors in Angiogenesis; Complex Partnerships. Cell. Mol. Life Sci..

[56] Leppänen V.M., Tvorogov D., Kisko K., Prota A.E., Jeltsch M., Anisimov A., Markovic-Mueller S., Stuttfeld E., Goldie K.N., Ballmer-Hofer K. (2013). Structural and Mechanistic Insights into VEGF Receptor 3 Ligand Binding and Activation. Proc. Natl. Acad. Sci. USA.

[57] Stuttfeld E., Ballmer-Hofer K. (2009). Structure and Function of VEGF Receptors. IUBMB Life.

[58] Fuh G., Li B., Crowley C., Cunningham B., Wells J.A. (1998). Requirements for Binding and Signaling of the Kinase Domain Receptor for Vascular Endothelial Growth Factor. J. Biol. Chem..

[59] Takahashi H., Shibuya M. (2005). The Vascular Endothelial Growth Factor (VEGF)/VEGF Receptor System and Its Role under Physiological and Pathological Conditions. Clin. Sci..

[60] Simons M., Gordon E., Claesson-Welsh L. (2016). Mechanisms and Regulation of Endothelial VEGF Receptor Signalling. Nat. Rev. Mol. Cell Biol..

[61] Tammela T., Zarkada G., Wallgard E., Murtomäki A., Suchting S., Wirzenius M., Waltari M., Hellström M., Schomber T., Peltonen R. (2008). Blocking VEGFR-3 Suppresses Angiogenic Sprouting and Vascular Network Formation. Nature.

[62] Jena M.K., Sharma N.R., Petitt M., Maulik D., Nayak N.R. (2020). Pathogenesis of Preeclampsia and Therapeutic Approaches Targeting the Placenta. Biomolecules.

[63] Goldman C.K., Kendall R.L., Cabrera G., Soroceanu L., Heike Y., Gillespie G.Y., Siegal G.P., Mao X., Bett A.J., Huckle W.R. (1998). Paracrine Expression of a Native Soluble Vascular Endothelial Growth Factor Receptor Inhibits Tumor Growth, Metastasis, and Mortality Rate. Proc. Natl. Acad. Sci. USA.

[64] Ikeda T., Saito-Takatsuji H., Yoshitomi Y., Yonekura H. (2020). Role of Arginine Methylation in Alternative Polyadenylation of VEGFR-1 (Flt-1) Pre-MRNA. Int. J. Mol. Sci..

[65] Rahimi N. (2006). Vascular Endothelial Growth Factor Receptors: Molecular Mechanisms of Activation and Therapeutic Potentials. Exp. Eye Res..

[66] Rahimi N. (2006). VEGFR-1 and VEGFR-2: Two Non-Identical Twins with a Unique Physiognomy. Front. Biosci..

[67] Tjwa M., Luttun A., Autiero M., Carmeliet P. (2003). VEGF and PlGF: Two Pleiotropic Growth Factors with Distinct Roles in Development and Homeostasis. Cell Tissue Res..

[68] Geretti E., Klagsbrun M. (2007). Neuropilins: Novel Targets for Anti-Angiogenesis Therapies. Cell Adh. Migr..

[69] Niland S., Eble J.A. (2019). Neuropilins in the Context of Tumor Vasculature. Int. J. Mol. Sci..

[70] Dumbrăveanu L., Cușnir V., Bobescu D. (2021). A Review of Neovascular Glaucoma. Etiopathogenesis and Treatment. Rom. J. Ophthalmol..

[71] Gomułka K., Liebhart J., Gładysz U., Mȩdrala W. (2019). VEGF Serum Concentration and Irreversible Bronchoconstriction in Adult Asthmatics. Adv. Clin. Exp. Med..

[72] Hoshino M., Nakamura Y., Hamid Q.A. (2001). Gene Expression of Vascular Endothelial Growth Factor and Its Receptors and Angiogenesis in Bronchial Asthma. J. Allergy Clin. Immunol..

[73] Meyer N., Christoph J., Makrinioti H., Indermitte P., Rhyner C., Soyka M., Eiwegger T., Chalubinski M., Wanke K., Fujita H. (2012). Inhibition of Angiogenesis by IL-32: Possible Role in Asthma. J. Allergy Clin. Immunol..

[74] Lopez-Guisa J.M., Powers C., File D., Cochrane E., Jimenez N., Debley J.S. (2012). Airway Epithelial Cells from Asthmatic Children Differentially Express Proremodeling Factors. J. Allergy Clin. Immunol..

[75] Hoshino M., Takahashi M., Aoike N. (2001). Expression of Vascular Endothelial Growth Factor, Basic Fibroblast Growth Factor, and Angiogenin Immunoreactivity in Asthmatic Airways and Its Relationship to Angiogenesis. J. Allergy Clin. Immunol..

[76] Zhang Y., Lin J., Ren Z., Li X. (2018). Predictive Effect of Exhaled NO and VEGF Expression Levels on the Severity of Bronchial Asthma and Airway Inflammation. Int. J. Clin. Exp. Pathol..

[77] Wang K., Liu C.T., Wu Y.H., Feng Y.L., Bai H.L. (2008). Budesonide/Formoterol Decreases Expression of Vascular Endothelial Growth Factor (VEGF) and VEGF Receptor 1 within Airway Remodelling in Asthma. Adv. Ther..

[78] Kim S.H., Pei Q.M., Jiang P., Liu J., Sun R.F., Qian X.J., Liu J.B. (2019). Upregulation of MUC5AC by VEGF in Human Primary Bronchial Epithelial Cells: Implications for Asthma. Respir. Res..

[79] Kim S.H., Pei Q.M., Jiang P., Yang M., Qian X.J., Liu J.B. (2017). Role of Licochalcone A in VEGF-Induced Proliferation of Human Airway Smooth Muscle Cells: Implications for Asthma. Growth Factors.

[80] Wang H., Keiser J.A. (1998). Vascular Endothelial Growth Factor Upregulates the Expression of Matrix Metalloproteinases in Vascular Smooth Muscle Cells: Role of Flt-1. Circ. Res..

[81] Pei Q.M., Jiang P., Yang M., Qian X.J., Liu J.B., Zheng H., Zhao L.H., Kim S.H. (2016). Upregulation of a Disintegrin and Metalloproteinase-33 by VEGF in Human Airway Smooth Muscle Cells: Implications for Asthma. Cell Cycle.

[82] Clifford R.L., John A.E., Brightling C.E., Knox A.J. (2012). Abnormal Histone Methylation Is Responsible for Increased Vascular Endothelial Growth Factor 165a Secretion from Airway Smooth Muscle Cells in Asthma. J. Immunol..

[83] Fujita H., Chalubinski M., Rhyner C., Indermitte P., Meyer N., Ferstl R., Treis A., Gomez E., Akkaya A., O’Mahony L. (2011). Claudin-1 Expression in Airway Smooth Muscle Exacerbates Airway Remodeling in Asthmatic Subjects. J. Allergy Clin. Immunol..

[84] de Paulis A., Prevete N., Fiorentino I., Rossi F.W., Staibano S., Montuori N., Ragno P., Longobardi A., Liccardo B., Genovese A. (2006). Expression and Functions of the Vascular Endothelial Growth Factors and Their Receptors in Human Basophils. J. Immunol..

[85] Sumbayev V.V., Nicholas S.A., Streatfield C.L., Gibbs B.F. (2009). Involvement of Hypoxia-Inducible Factor-1 HiF(1alpha) in IgE-Mediated Primary Human Basophil Responses. Eur. J. Immunol..

[86] Corrigan C.J., Wang W., Meng Q., Fang C., Wu H., Reay V., Lv Z., Fan Y., An Y., Wang Y.H. (2011). T-Helper Cell Type 2 (Th2) Memory T Cell-Potentiating Cytokine IL-25 Has the Potential to Promote Angiogenesis in Asthma. Proc. Natl. Acad. Sci. USA.

[87] Bucher F., Lee J., Shin S., Kim M.S., Oh Y.S., Ha S., Zhang H., Yea K. (2018). Interleukin-5 Suppresses Vascular Endothelial Growth Factor-Induced Angiogenesis through STAT5 Signaling. Cytokine.

[88] Liu J., Wang W., Wang L., Chen S., Tian B., Huang K., Corrigan C.J., Ying S., Wang W., Wang C. (2018). IL-33 Initiates Vascular Remodelling in Hypoxic Pulmonary Hypertension by up-Regulating HIF-1α and VEGF Expression in Vascular Endothelial Cells. EBioMedicine.

[89] Lee C.G., Link H., Baluk P., Homer R.J., Chapoval S., Bhandari V., Kang M.J., Cohn L., Kim Y.K., McDonald D.M. (2004). Vascular Endothelial Growth Factor (VEGF) Induces Remodeling and Enhances TH2-Mediated Sensitization and Inflammation in the Lung. Nat. Med..

[90] Meyer N., Akdis C.A. (2013). Vascular Endothelial Growth Factor as a Key Inducer of Angiogenesis in the Asthmatic Airways. Curr. Allergy Asthma Rep..

[91] Psarras S., Volonaki E., Skevaki C.L., Xatzipsalti M., Bossios A., Pratsinis H., Tsigkos S., Gourgiotis D., Constantopoulos A.G., Papapetropoulos A. (2006). Vascular Endothelial Growth Factor-Mediated Induction of Angiogenesis by Human Rhinoviruses. J. Allergy Clin. Immunol..

[92] De Silva D., Dagher H., Ghildyal R., Lindsay M., Li X., Freezer N.J., Wilson J.W., Bardin P.G. (2006). Vascular Endothelial Growth Factor Induction by Rhinovirus Infection. J. Med. Virol..

[93] Lu H.Y., Zhao G.L., Fu M.F. (2016). Polymorphisms in the Vascular Endothelial Growth Factor (VEGF) Gene Associated with Asthma. Genet. Mol. Res..

[94] Sharma S., Murphy A.J., Soto-Quiros M.E., Avila L., Klanderman B.J., Sylvia J.S., Celedón J.C., Raby B.A., Weiss S.T. (2009). Association of VEGF Polymorphisms with Childhood Asthma, Lung Function and Airway Responsiveness. Eur. Respir. J..

[95] Simpson A., Custovic A., Tepper R., Graves P., Stern D.A., Jones M., Hankinson J., Curtin J.A., Wu J., Blekic M. (2012). Genetic Variation in Vascular Endothelial Growth Factor-a and Lung Function. Am. J. Respir. Crit. Care Med..

[96] Balantic M., Rijavec M., Kavalar M.S., Suskovic S., Silar M., Kosnik M., Korosec P. (2012). Asthma Treatment Outcome in Children Is Associated with Vascular Endothelial Growth Factor A (VEGFA) Polymorphisms. Mol. Diagn. Ther..

[97] Boix E., Nogués M.V. (2007). Mammalian Antimicrobial Proteins and Peptides: Overview on the RNase A Superfamily Members Involved in Innate Host Defence. Mol. Biosyst..

[98] Rosenberg H.F., Ackerman S.J., Tenen D.G. (1989). Human Eosinophil Cationic Protein. Molecular Cloning of a Cytotoxin and Helminthotoxin with Ribonuclease Activity. J. Exp. Med..

[99] Rosenberg H.F. (2008). RNase A Ribonucleases and Host Defense: An Evolving Story. J. Leukoc. Biol..

[100] Hogan S.P., Rosenberg H.F., Moqbel R., Phipps S., Foster P.S., Lacy P., Kay A.B., Rothenberg M.E. (2008). Eosinophils: Biological Properties and Role in Health and Disease. Clin. Exp. Allergy.

[101] Sur S., Glitz D.G., Kita H., Kujawa S.M., Peterson E.A., Weiler D.A., Kephart G.M., Wagner J.M., George T.J., Gleich G.J. (1998). Localization of Eosinophil-Derived Neurotoxin and Eosinophil Cationic Protein in Neutrophilic Leukocytes. J. Leukoc. Biol..

[102] Yang D., Rosenberg H.F., Chen Q., Dyer K.D., Kurosaka K., Oppenheim J.J. (2003). Eosinophil-Derived Neurotoxin (EDN), an Antimicrobial Protein with Chemotactic Activities for Dendritic Cells. Blood.

[103] Domachowske J.B., Dyer K.D., Bonville C.A., Rosenberg H.F. (1998). Recombinant Human Eosinophil-Derived Neurotoxin/RNase 2 Functions as an Effective Antiviral Agent against Respiratory Syncytial Virus. J. Infect. Dis..

[104] Rosenberg H.F., Domachowske J.B. (2001). Eosinophils, Eosinophil Ribonucleases, and Their Role in Host Defense against Respiratory Virus Pathogens. J. Leukoc. Biol..

[105] Harrison A.M., Bonville C.A., Rosenberg H.F., Domachowske J.B. (1999). Respiratory Syncytical Virus-Induced Chemokine Expression in the Lower Airways: Eosinophil Recruitment and Degranulation. Am. J. Respir. Crit. Care Med..

[106] Dosanjh A. (2020). Eosinophil-Derived Neurotoxin and Respiratory Tract Infection and Inflammation: Implications for COVID-19 Management. J. Interferon Cytokine Res..

[107] Dill-McFarland K.A., Schwartz J.T., Zhao H., Shao B., Fulkerson P.C., Altman M.C., Gill M.A. (2022). Eosinophil-Mediated Suppression and Anti-IL-5 Enhancement of Plasmacytoid Dendritic Cell Interferon Responses in Asthma. J. Allergy Clin. Immunol..

[108] Phipps S., Howard D.R., Werder R.B. (2022). Eosinophils Apply a Handbrake to Plasmacytoid Dendritic Cell Antiviral Immunity in Asthma. J. Allergy Clin. Immunol..

[109] Rosenberg H.F., Dyer K.D., Foster P.S. (2013). Eosinophils: Changing Perspectives in Health and Disease. Nat. Rev. Immunol..

[110] Yang D., Chen Q., Rosenberg H.F., Rybak S.M., Newton D.L., Wang Z.Y., Fu Q., Tchernev V.T., Wang M., Schweitzer B. (2004). Human Ribonuclease A Superfamily Members, Eosinophil-Derived Neurotoxin and Pancreatic Ribonuclease, Induce Dendritic Cell Maturation and Activation. J. Immunol..

[111] Yang D., Chen Q., Shao B.S., Zhang P., Kurosaka K., Caspi R.R., Michalek S.M., Rosenberg H.F., Zhang N., Oppenheim J.J. (2008). Eosinophil-Derived Neurotoxin Acts as an Alarmin to Activate the TLR2-MyD88 Signal Pathway in Dendritic Cells and Enhances Th2 Immune Responses. J. Exp. Med..

[112] Oppenheim J.J., Yang D. (2005). Alarmins: Chemotactic Activators of Immune Responses. Curr. Opin. Immunol..

[113] Yang D., Han Z., Oppenheim J.J. (2017). Alarmins and Immunity. Immunol. Rev..

[114] Kim C.K., Callaway Z., Park J.S., Kwon E. (2017). Utility of Serum Eosinophil-Derived Neurotoxin (EDN) Measurement by ELISA in Young Children with Asthma. Allergol. Int..

[115] Venge P. (2004). Monitoring the Allergic Inflammation. Allergy.

[116] Quoc Q.L., Moon J.Y., Lee D.H., Ban G.Y., Kim S.H., Park H.S. (2022). Role of Thymus and Activation-Regulated Chemokine in Allergic Asthma. J. Asthma Allergy.

[117] Yancey S.W., Keene O.N., Albers F.C., Ortega H., Bates S., Bleecker E.R., Pavord I. (2017). Biomarkers for Severe Eosinophilic Asthma. J. Allergy Clin. Immunol..

[118] Nelson R.K., Bush A., Stokes J., Nair P., Akuthota P. (2020). Eosinophilic Asthma. J. allergy Clin. Immunol. Pract..

[119] Crimi E., Spanevello A., Neri M., Ind P.W., Rossi G.A., Brusasco V. (1998). Dissociation between Airway Inflammation and Airway Hyperresponsiveness in Allergic Asthma. Am. J. Respir. Crit. Care Med..

[120] Jatakanon A., Lim S., Barnes P.J. (2000). Changes in Sputum Eosinophils Predict Loss of Asthma Control. Am. J. Respir. Crit. Care Med..

[121] An J., Lee J.H., Sim J.H., Song W.J., Kwon H.S., Cho Y.S., Moon H.B., Kim C.K., Kim T.B. (2020). Serum Eosinophil-Derived Neurotoxin Better Reflect Asthma Control Status Than Blood Eosinophil Counts. J. Allergy Clin. Immunol. Pract..

[122] Kim C.K., Kita H., Callaway Z., Kim H.B., Choi J., Fujisawa T., Shin B.M., Koh Y.Y. (2010). The Roles of a Th2 Cytokine and CC Chemokine in Children with Stable Asthma: Potential Implication in Eosinophil Degranulation. Pediatr. Allergy Immunol..

[123] Kim C.K., Callaway Z., Kim D.W., Kita H. (2011). Eosinophil Degranulation Is More Important than Eosinophilia in Identifying Asthma in Chronic Cough. J. Asthma.

[124] Lee Y., Lee J.H., Yang E.M., Kwon E.M., Jung C.G., Kim S.C., Choi Y., Cho Y.S., Kim C.K., Park H.S. (2019). Serum Levels of Eosinophil-Derived Neurotoxin: A Biomarker for Asthma Severity in Adult Asthmatics. Allergy Asthma Immunol. Res..

[125] Soo Kim H., Yang H.J., Song D.J., Ju Lee Y., In Suh D., Yeon Shim J., Yoo Y., Keun Kim C., Min Ahn Y., Tack Kim J. (2022). Eosinophil-Derived Neurotoxin: An Asthma Exacerbation Biomarker in Children. Allergy Asthma Proc..

[126] Dharmage S.C., Perret J.L., Custovic A. (2019). Epidemiology of Asthma in Children and Adults. Front. Pediatr..

[127] Kim C.K., Seo J.K., Ban S.H., Fujisawa T., Kim D.W., Callaway Z. (2013). Eosinophil-Derived Neurotoxin Levels at 3 Months Post-Respiratory Syncytial Virus Bronchiolitis Are a Predictive Biomarker of Recurrent Wheezing. Biomarkers.

[128] Granger V., Zerimech F., Arab J., Siroux V., De Nadai P., Tsicopoulos A., Matran R., Akiki Z., Nadif R. (2022). Blood Eosinophil Cationic Protein and Eosinophil-Derived Neurotoxin Are Associated with Different Asthma Expression and Evolution in Adults. Thorax.

[129] Gon Y., Ito R., Hattori T., Hiranuma H., Kumasawa F., Kozu Y., Endo D., Koyama D., Shintani Y., Eriko T. (2015). Serum Eosinophil-Derived Neurotoxin: Correlation with Persistent Airflow Limitation in Adults with House-Dust Mite Allergic Asthma. Allergy Asthma Proc..

[130] Bellin M.H., Osteen P., Kub J., Bollinger M.E., Tsoukleris M., Chaikind L., Butz A.M. (2015). Stress and Quality of Life in Urban Caregivers of Children With Poorly Controlled Asthma: A Longitudinal Analysis. J. Pediatr. Health Care.

[131] Hoch H.E., Houin P.R., Stillwell P.C. (2019). Asthma in Children: A Brief Review for Primary Care Providers. Pediatr. Ann..

[132] Del Rio P.R., Liu A.H., Borres M.P., Södergren E., Iachetti F., Casale T.B. (2022). Asthma and Allergy: Unravelling a Tangled Relationship with a Focus on New Biomarkers and Treatment. Int. J. Mol. Sci..

[133] Kim C.K., Callaway Z., Park J.S., Nishimori H., Ogino T., Nagao M., Fujisawa T. (2018). Montelukast Reduces Serum Levels of Eosinophil-Derived Neurotoxin in Preschool Asthma. Allergy Asthma Immunol. Res..

[134] Kim K.W., Lee K.E., Kim E.S., Song T.W., Sohn M.H., Kim K.E. (2007). Serum Eosinophil-Derived Neurotoxin (EDN) in Diagnosis and Evaluation of Severity and Bronchial Hyperresponsiveness in Childhood Asthma. Lung.

[135] Hirst S.J., Lee T.H. (1998). Airway Smooth Muscle as a Target of Glucocorticoid Action in the Treatment of Asthma. Am. J. Respir. Crit. Care Med..

[136] Ora J., Calzetta L., Matera M.G., Cazzola M., Rogliani P. (2020). Advances with Glucocorticoids in the Treatment of Asthma: State of the Art. Expert Opin. Pharmacother..

[137] Wen F.Q., Liu X., Manda W., Terasaki Y., Kobayashi T., Abe S., Fang Q., Ertl R., Manouilova L., Rennard S.I. (2003). TH2 Cytokine-Enhanced and TGF-β-Enhanced Vascular Endothelial Growth Factor Production by Cultured Human Airway Smooth Muscle Cells Is Attenuated by IFN-γ and Corticosteroids. J. Allergy Clin. Immunol..

[138] Skevaki C.L., Christodoulou I., Spyridaki I.S., Tiniakou I., Georgiou V., Xepapadaki P., Kafetzis D.A., Papadopoulos N.G. (2009). Budesonide and Formoterol Inhibit Inflammatory Mediator Production by Bronchial Epithelial Cells Infected with Rhinovirus. Clin. Exp. Allergy.

[139] Hamamoto Y., Ano S., Allard B., O’Sullivan M., McGovern T.K., Martin J.G. (2017). Montelukast Reduces Inhaled Chlorine Triggered Airway Hyperresponsiveness and Airway Inflammation in the Mouse. Br. J. Pharmacol..

[140] Türkeli A., Yilmaz Ö., Karaman M., Kanik E., Firinci F., İnan S., Yüksel H. (2021). Anti-VEGF Treatment Suppresses Remodeling Factors and Restores Epithelial Barrier Function through the E-Cadherin/β-Catenin Signaling Axis in Experimental Asthma Models. Exp. Ther. Med..

[141] Ge A., Liu Y., Zeng X., Kong H., Ma Y., Zhang J., Bai F., Huang M. (2015). Effect of Diosmetin on Airway Remodeling in a Murine Model of Chronic Asthma. Acta Biochim. Biophys. Sin..

[142] Huang C., Dong H., Zou M., Luo L., Hu Y., Xie Z., Le Y., Liu L., Zou F., Cai S. (2016). Bevacizumab Reduced Auto-Phosphorylation of VEGFR2 to Protect HDM-Induced Asthma Mice. Biochem. Biophys. Res. Commun..

[143] Bolandi S.M., Abdolmaleki Z., Assarehzadegan M.A. (2021). Anti-Angiogenic Properties of Bevacizumab Improve Respiratory System Inflammation in Ovalbumin-Induced Rat Model of Asthma. Inflammation.

[144] Jang S., Park J.W., Cha H.R., Jung S.Y., Lee J.E., Jung S.S., Kim J.O., Kim S.Y., Lee C.S., Park H.S. (2012). Silver Nanoparticles Modify VEGF Signaling Pathway and Mucus Hypersecretion in Allergic Airway Inflammation. Int. J. Nanomed..

[145] Kalishwaralal K., Banumathi E., Pandian S.B.R.K., Deepak V., Muniyandi J., Eom S.H., Gurunathan S. (2009). Silver Nanoparticles Inhibit VEGF Induced Cell Proliferation and Migration in Bovine Retinal Endothelial Cells. Colloids Surf. B. Biointerfaces.

[146] Zha W., Su M., Huang M., Cai J., Du Q. (2016). Administration of Pigment Epithelium-Derived Factor Inhibits Airway Inflammation and Remodeling in Chronic OVA-Induced Mice via VEGF Suppression. Allergy Asthma Immunol. Res..

[147] Kim S.R., Lee K.S., Lee K.B., Lee Y.C. (2012). Recombinant IGFBP-3 Inhibits Allergic Lung Inflammation, VEGF Production, and Vascular Leak in a Mouse Model of Asthma. Allergy.

[148] Yang N., Li X. (2022). Epigallocatechin Gallate Relieves Asthmatic Symptoms in Mice by Suppressing HIF-1α/VEGFA-Mediated M2 Skewing of Macrophages. Biochem. Pharmacol..

[149] Li Y.Z., Chen L.X., Guo F.F., Cao Y., Hu W., Shi Y., Lin X.C., Hou J., Li L.P., Ding X.F. (2019). Effects of Epigallocatechin-3-Gallate on the HMGB1/RAGE Pathway in PM2.5-Exposed Asthmatic Rats. Biochem. Biophys. Res. Commun..

[150] Zhao X., Yu F.Q., Huang X.J., Xu B.Y., Li Y.L., Zhao X.Y., Guo H.F., Luan B. (2018). Azithromycin Influences Airway Remodeling in Asthma via the PI3K/Akt/MTOR/HIF-1α/VEGF Pathway. J. Biol. Regul. Homeost. Agents.

[151] Willems-Widyastuti A., Vanaudenaerde B.M., Vos R., Dilisen E., Verleden S.E., De Vleeschauwer S.I., Vaneylen A., Mooi W.J., de Boer W.I., Sharma H.S. (2013). Azithromycin Attenuates Fibroblast Growth Factors Induced Vascular Endothelial Growth Factor via P38(MAPK) Signaling in Human Airway Smooth Muscle Cells. Cell Biochem. Biophys..

[152] Gibson P.G., Yang I.A., Upham J.W., Reynolds P.N., Hodge S., James A.L., Jenkins C., Peters M.J., Marks G.B., Baraket M. (2019). Efficacy of Azithromycin in Severe Asthma from the AMAZES Randomised Trial. ERJ Open Res..

[153] Ghimire J.J., Jat K.R., Sankar J., Lodha R., Iyer V.K., Gautam H., Sood S., Kabra S.K. (2022). Azithromycin for Poorly Controlled Asthma in Children: A Randomized Controlled Trial. Chest.

[154] Kim S.H., Pei Q.M., Jiang P., Yang M., Qian X.J., Liu J.B. (2017). Effect of Active Vitamin D3 on VEGF-Induced ADAM33 Expression and Proliferation in Human Airway Smooth Muscle Cells: Implications for Asthma Treatment. Respir. Res..

[155] Lee S.Y., Chung S.M. (2007). Neovastat (AE-941) Inhibits the Airway Inflammation via VEGF and HIF-2 Alpha Suppression. Vascul. Pharmacol..

[156] Suzaki Y., Hamada K., Sho M., Ito T., Miyamoto K., Akashi S., Kashizuka H., Ikeda N., Nakajima Y., Iwase M. (2005). A Potent Antiangiogenic Factor, Endostatin Prevents the Development of Asthma in a Murine Model. J. Allergy Clin. Immunol..

[157] Yu Q., Yu X., Zhong X., Ma Y., Wu Y., Bian T., Huang M., Zeng X. (2020). Melatonin Modulates Airway Smooth Muscle Cell Phenotype by Targeting the STAT3/Akt/GSK-3β Pathway in Experimental Asthma. Cell Tissue Res..

[158] Kips J.C., O’Connor B.J., Inman M.D., Svensson K., Pauwels R.A., O’Byrne P.M. (2000). A Long-Term Study of the Antiinflammatory Effect of Low-Dose Budesonide plus Formoterol versus High-Dose Budesonide in Asthma. Am. J. Respir. Crit. Care Med..

[159] Nicholas T.P., Haick A.K., Bammler T.K., Workman T.W., Kavanagh T.J., Faustman E.M., Gharib S.A., Altemeier W.A. (2020). The Effects of Genotype × Phenotype Interactions on Transcriptional Response to Silver Nanoparticle Toxicity in Organotypic Cultures of Murine Tracheal Epithelial Cells. Toxicol. Sci..

[160] Taniguchi H., Tsuchida T., Nakamura Y., Motoshima K., Mizoguchi K., Kohno S. (2013). Severe, but Manageable Hypoxia Caused by Bronchospasm Induced by Bevacizumab. Respirol. Case Rep..

[161] Parnham M.J., Haber V.E., Giamarellos-Bourboulis E.J., Perletti G., Verleden G.M., Vos R. (2014). Azithromycin: Mechanisms of Action and Their Relevance for Clinical Applications. Pharmacol. Ther..

[162] Gibson P.G., Yang I.A., Upham J.W., Reynolds P.N., Hodge S., James A.L., Jenkins C., Peters M.J., Marks G.B., Baraket M. (2017). Effect of Azithromycin on Asthma Exacerbations and Quality of Life in Adults with Persistent Uncontrolled Asthma (AMAZES): A Randomised, Double-Blind, Placebo-Controlled Trial. Lancet.

[163] Ge A., Ma Y., Liu Y.N., Li Y.S., Gu H., Zhang J.X., Wang Q.X., Zeng X.N., Huang M. (2016). Diosmetin Prevents TGF-Β1-Induced Epithelial-Mesenchymal Transition via ROS/MAPK Signaling Pathways. Life Sci..

[164] Villa P., Cova D., De Francesco L., Guaitani A., Palladini G., Perego R. (1992). Protective Effect of Diosmetin on in Vitro Cell Membrane Damage and Oxidative Stress in Cultured Rat Hepatocytes. Toxicology.

[165] Forno E., Bacharier L.B., Phipatanakul W., Guilbert T.W., Cabana M.D., Ross K., Covar R., Gern J.E., Rosser F.J., Blatter J. (2020). Effect of Vitamin D3 Supplementation on Severe Asthma Exacerbations in Children With Asthma and Low Vitamin D Levels: The VDKA Randomized Clinical Trial. JAMA.

[166] Jiao J., King T.S., McKenzie M., Bacharier L.B., Dixon A.E., Codispoti C.D., Dunn R.M., Grossman N.L., Lugogo N.L., Ramratnam S.K. (2016). Vitamin D3 Therapy in Patients with Asthma Complicated by Sinonasal Disease: Secondary Analysis of the Vitamin D Add-on Therapy Enhances Corticosteroid Responsiveness in Asthma Trial. J. Allergy Clin. Immunol..

[167] Shan L., Kang X., Liu F., Cai X., Han X., Shang Y. (2018). Epigallocatechin Gallate Improves Airway Inflammation through TGF-β1 Signaling Pathway in Asthmatic Mice. Mol. Med. Rep..

[168] Yang N., Zhang H., Cai X., Shang Y. (2018). Epigallocatechin-3-Gallate Inhibits Inflammation and Epithelial-mesenchymal Transition through the PI3K/AKT Pathway via Upregulation of PTEN in Asthma. Int. J. Mol. Med..

[169] Lee H., Kim S.R., Oh Y., Cho S.H., Schleimer R.P., Lee Y.C. (2014). Targeting Insulin-like Growth Factor-I and Insulin-like Growth Factor-Binding Protein-3 Signaling Pathways. A Novel Therapeutic Approach for Asthma. Am. J. Respir. Cell Mol. Biol..

[170] Lee S.Y., Paik S.Y., Chung S.M. (2005). Neovastat (AE-941) Inhibits the Airway Inflammation and Hyperresponsiveness in a Murine Model of Asthma. J. Microbiol..

[171] Shin I.S., Jeon W.Y., Shin H.K., Lee M.Y. (2013). Effects of Montelukast on Subepithelial/Peribronchial Fibrosis in a Murine Model of Ovalbumin Induced Chronic Asthma. Int. Immunopharmacol..

[172] Calapai G., Casciaro M., Miroddi M., Calapai F., Navarra M., Gangemi S. (2014). Montelukast-Induced Adverse Drug Reactions: A Review of Case Reports in the Literature. Pharmacology.

[173] Ek E.T.H., Dass C.R., Choong P.F.M. (2006). PEDF: A Potential Molecular Therapeutic Target with Multiple Anti-Cancer Activities. Trends Mol. Med..

[174] Chen Y., Wu X., Yang X., Liu X., Zeng Y., Li J. (2021). Melatonin Antagonizes Ozone-Exacerbated Asthma by Inhibiting the TRPV1 Channel and Stabilizing the Nrf2 Pathway. Environ. Sci. Pollut. Res. Int..

[175] Shin I.S., Park J.W., Shin N.R., Jeon C.M., Kwon O.K., Lee M.Y., Kim H.S., Kim J.C., Oh S.R., Ahn K.S. (2014). Melatonin Inhibits MUC5AC Production via Suppression of MAPK Signaling in Human Airway Epithelial Cells. J. Pineal Res..

[176] Bel E.H., ten Brinke A. (2017). New Anti-Eosinophil Drugs for Asthma and COPD: Targeting the Trait!. Chest.

[177] Jang J.H., Woo S.D., Lee Y., Kim C.K., Shin Y.S., Ye Y.M., Park H.S. (2021). Changes in Type 2 Biomarkers After Anti-IL5 Treatment in Patients With Severe Eosinophilic Asthma. Allergy Asthma Immunol. Res..

[178] Nair P., Ochkur S.I., Protheroe C., Radford K., Efthimiadis A., Lee N.A., Lee J.J. (2013). Eosinophil Peroxidase in Sputum Represents a Unique Biomarker of Airway Eosinophilia. Allergy.

[179] Rutten B., Young S., Rhedin M., Olsson M., Kurian N., Syed F., Beech A., Fidock M., Newbold P., Singh D. (2021). Eosinophil-Derived Neurotoxin: A Biologically and Analytically Attractive Asthma Biomarker. PLoS ONE.

[180] Agache I., Beltran J., Akdis C., Akdis M., Canelo-Aybar C., Canonica G.W., Casale T., Chivato T., Corren J., Del Giacco S. (2020). Efficacy and Safety of Treatment with Biologicals (Benralizumab, Dupilumab, Mepolizumab, Omalizumab and Reslizumab) for Severe Eosinophilic Asthma. A Systematic Review for the EAACI Guidelines—Recommendations on the Use of Biologicals in Severe Asthma. Allergy.

[181] Pham T.H., Damera G., Newbold P., Ranade K. (2016). Reductions in Eosinophil Biomarkers by Benralizumab in Patients with Asthma. Respir. Med..

[182] Haktanir Abul M., Phipatanakul W. (2019). Severe Asthma in Children: Evaluation and Management. Allergol. Int..

[183] Kelly E.A.B., Busse W.W., Jarjour N.N. (2000). Inhaled Budesonide Decreases Airway Inflammatory Response to Allergen. Am. J. Respir. Crit. Care Med..

[184] Pedersen B., Dahl R., Karlström R., Peterson C.G.B., Venge P. (1996). Eosinophil and Neutrophil Activity in Asthma in a One-Year Trial with Inhaled Budesonide. The Impact of Smoking. Am. J. Respir. Crit. Care Med..

[185] Chipps B.E., Albers F.C., Reilly L., Johnsson E., Cappelletti C., Papi A. (2021). Efficacy and Safety of As-Needed Albuterol/Budesonide versus Albuterol in Adults and Children Aged ≥4 Years with Moderate-to-Severe Asthma: Rationale and Design of the Randomised, Double-Blind, Active-Controlled MANDALA Study. BMJ Open Respir. Res..

[186] Kavanagh J.E., Hearn A.P., Dhariwal J., d’Ancona G., Douiri A., Roxas C., Fernandes M., Green L., Thomson L., Nanzer A.M. (2021). Real-World Effectiveness of Benralizumab in Severe Eosinophilic Asthma. Chest.

[187] Makiya M.A., Khoury P., Kuang F.L., Mata A.D., Mahmood S., Bowman A., Espinoza D., Kovacs N., Brown T., Holland N. (2023). Urine Eosinophil-Derived Neurotoxin: A Potential Marker of Activity in Select Eosinophilic Disorders. Allergy.

[188] Guntur V.P., Manka L.A., Denson J.L., Dunn R.M., Dollin Y.T., Gill M., Kolakowski C., Strand M.J., Wechsler M.E. (2021). Benralizumab as a Steroid-Sparing Treatment Option in Eosinophilic Granulomatosis with Polyangiitis. J. Allergy Clin. Immunol. Pract..

[189] Korn S., Bourdin A., Chupp G., Cosio B.G., Arbetter D., Shah M., Gil E.G. (2021). Integrated Safety and Efficacy Among Patients Receiving Benralizumab for Up to 5 Years. J. Allergy Clin. Immunol. Pract..

[190] Kwah J.H., Peters A.T. (2019). Asthma in Adults: Principles of Treatment. Allergy Asthma Proc..

[191] Charles D., Shanley J., Temple S.N., Rattu A., Khaleva E., Roberts G. (2022). Real-World Efficacy of Treatment with Benralizumab, Dupilumab, Mepolizumab and Reslizumab for Severe Asthma: A Systematic Review and Meta-Analysis. Clin. Exp. Allergy.

[192] Chapman K.R., Albers F.C., Chipps B., Muñoz X., Devouassoux G., Bergna M., Galkin D., Azmi J., Mouneimne D., Price R.G. (2019). The Clinical Benefit of Mepolizumab Replacing Omalizumab in Uncontrolled Severe Eosinophilic Asthma. Allergy.

[193] Ortega H.G., Liu M.C., Pavord I.D., Brusselle G.G., FitzGerald J.M., Chetta A., Humbert M., Katz L.E., Keene O.N., Yancey S.W. (2014). Mepolizumab Treatment in Patients with Severe Eosinophilic Asthma. N. Engl. J. Med..

[194] Harvey E.S., Langton D., Katelaris C., Stevens S., Farah C.S., Gillman A., Harrington J., Hew M., Kritikos V., Radhakrishna N. (2020). Mepolizumab Effectiveness and Identification of Super-Responders in Severe Asthma. Eur. Respir. J..

[195] Pavord I.D., Korn S., Howarth P., Bleecker E.R., Buhl R., Keene O.N., Ortega H., Chanez P. (2012). Mepolizumab for Severe Eosinophilic Asthma (DREAM): A Multicentre, Double-Blind, Placebo-Controlled Trial. Lancet.

[196] Pérez de Llano L.A., Cosío B.G., Lobato Astiárraga I., Soto Campos G., Tejedor Alonso M.Á., Marina Malanda N., Padilla Galo A., Urrutia Landa I., Michel de la Rosa F.J., García-Moguel I. (2022). Asthma Control in Patients with Severe Eosinophilic Asthma Treated with Reslizumab: Spanish Real-Life Data. J. Asthma Allergy.

[197] Castro M., Zangrilli J., Wechsler M.E., Bateman E.D., Brusselle G.G., Bardin P., Murphy K., Maspero J.F., O’Brien C., Korn S. (2015). Reslizumab for Inadequately Controlled Asthma with Elevated Blood Eosinophil Counts: Results from Two Multicentre, Parallel, Double-Blind, Randomised, Placebo-Controlled, Phase 3 Trials. Lancet. Respir. Med..

[198] Wechsler M.E., Ford L.B., Maspero J.F., Pavord I.D., Papi A., Bourdin A., Watz H., Castro M., Nenasheva N.M., Tohda Y. (2022). Long-Term Safety and Efficacy of Dupilumab in Patients with Moderate-to-Severe Asthma (TRAVERSE): An Open-Label Extension Study. Lancet. Respir. Med..

[199] Bacharier L.B., Maspero J.F., Katelaris C.H., Fiocchi A.G., Gagnon R., de Mir I., Jain N., Sher L.D., Mao X., Liu D. (2021). Dupilumab in Children with Uncontrolled Moderate-to-Severe Asthma. N. Engl. J. Med..

[200] Dupin C., Belhadi D., Guilleminault L., Gamez A.S., Berger P., De Blay F., Bonniaud P., Leroyer C., Mahay G., Girodet P.O. (2020). Effectiveness and Safety of Dupilumab for the Treatment of Severe Asthma in a Real-Life French Multi-Centre Adult Cohort. Clin. Exp. Allergy.

[201] Harb H., Chatila T.A. (2020). Mechanisms of Dupilumab. Clin. Exp. Allergy.

[202] Szefler S.J., Roberts G., Rubin A.S., Zielen S., Kuna P., Alpan O., Anzures-Cabrera J., Chen Q., Holweg C.T.J., Kaminski J. (2022). Efficacy, Safety, and Tolerability of Lebrikizumab in Adolescent Patients with Uncontrolled Asthma (ACOUSTICS). Clin. Transl. Allergy.

[203] Austin C.D., Gonzalez Edick M., Ferrando R.E., Solon M., Baca M., Mesh K., Bradding P., Gauvreau G.M., Sumino K., FitzGerald J.M. (2020). A Randomized, Placebo-Controlled Trial Evaluating Effects of Lebrikizumab on Airway Eosinophilic Inflammation and Remodelling in Uncontrolled Asthma (CLAVIER). Clin. Exp. Allergy.

[204] Hanania N.A., Korenblat P., Chapman K.R., Bateman E.D., Kopecky P., Paggiaro P., Yokoyama A., Olsson J., Gray S., Holweg C.T.J. (2016). Efficacy and Safety of Lebrikizumab in Patients with Uncontrolled Asthma (LAVOLTA I and LAVOLTA II): Replicate, Phase 3, Randomised, Double-Blind, Placebo-Controlled Trials. Lancet. Respir. Med..

[205] Hanania N.A., Noonan M., Corren J., Korenblat P., Zheng Y., Fischer S.K., Cheu M., Putnam W.S., Murray E., Scheerens H. (2015). Lebrikizumab in Moderate-to-Severe Asthma: Pooled Data from Two Randomised Placebo-Controlled Studies. Thorax.

[206] Kardas G., Panek M., Kuna P., Damiański P., Kupczyk M. (2022). Monoclonal Antibodies in the Management of Asthma: Dead Ends, Current Status and Future Perspectives. Front. Immunol..

[207] Edris A., De Feyter S., Maes T., Joos G., Lahousse L. (2019). Monoclonal Antibodies in Type 2 Asthma: A Systematic Review and Network Meta-Analysis. Respir. Res..

[208] Piper E., Brightling C., Niven R., Oh C., Faggioni R., Poon K., She D., Kell C., May R.D., Geba G.P. (2013). A Phase II Placebo-Controlled Study of Tralokinumab in Moderate-to-Severe Asthma. Eur. Respir. J..

[209] Gon Y., Maruoka S., Mizumura K. (2022). Omalizumab and IgE in the Control of Severe Allergic Asthma. Front. Pharmacol..

[210] Kotoulas S.C., Tsiouprou I., Fouka E., Pataka A., Papakosta D., Porpodis K. (2022). Omalizumab: An Optimal Choice for Patients with Severe Allergic Asthma. J. Pers. Med..

[211] Hanania N.A., Alpan O., Hamilos D.L., Condemi J.J., Reyes-Rivera I., Zhu J., Rosen K.E., Eisner M.D., Wong D.A., Busse W. (2011). Omalizumab in Severe Allergic Asthma Inadequately Controlled with Standard Therapy: A Randomized Trial. Ann. Intern. Med..

[212] Dorey-Stein Z.L., Shenoy K.V. (2021). Tezepelumab as an Emerging Therapeutic Option for the Treatment of Severe Asthma: Evidence to Date. Drug Des. Devel. Ther..

[213] Menzies-Gow A., Corren J., Bourdin A., Chupp G., Israel E., Wechsler M.E., Brightling C.E., Griffiths J.M., Hellqvist Å., Bowen K. (2021). Tezepelumab in Adults and Adolescents with Severe, Uncontrolled Asthma. N. Engl. J. Med..

[214] Jahangir A., Sattar S.B.A., Niazi M.R.K., Muhammad M., Jahangir A., Sahra S., Sharif M.A., Anwar M.Y., Chalhoub M. (2022). Efficacy and Safety of Fevipiprant in Asthma: A Review and Meta-Analysis. Cureus.

[215] Wojtukiewicz M.Z., Mysliwiec M., Sierko E., Sobierska M., Kruszewska J., Lipska A., Radziwon P., Tucker S.C., Honn K.V. (2020). Elevated Microparticles, Thrombin-Antithrombin and VEGF Levels in Colorectal Cancer Patients Undergoing Chemotherapy. Pathol. Oncol. Res..

[216] Song M., Wang H., Ye Q. (2020). Increased Circulating Vascular Endothelial Growth Factor in Acute Myeloid Leukemia Patients: A Systematic Review and Meta-Analysis. Syst. Rev..

[217] Filipiak J., Boinska J., Ziołkowska K., Zduńska M., Zarychta E., Rość D. (2021). Assessment of Endothelial Progenitor Cells, VEGF-A and SDF-1α in Hodgkin’s Lymphoma. Blood Coagul. Fibrinolysis.

[218] Botelho F., Pina F., Lunet N. (2010). VEGF and Prostatic Cancer: A Systematic Review. Eur. J. Cancer Prev..

[219] Yuan Q., Sun L., Li J.J., An C.H. (2014). Elevated VEGF Levels Contribute to the Pathogenesis of Osteoarthritis. BMC Musculoskelet. Disord..

[220] Marneros A.G. (2016). Increased VEGF-A Promotes Multiple Distinct Aging Diseases of the Eye through Shared Pathomechanisms. EMBO Mol. Med..

[221] Shim J.W., Madsen J.R. (2018). VEGF Signaling in Neurological Disorders. Int. J. Mol. Sci..

[222] Zhou W., Liu K., Zeng L., He J., Gao X., Gu X., Chen X., Jing Li J., Wang M., Wu D. (2022). Targeting VEGF-A/VEGFR2 Y949 Signaling-Mediated Vascular Permeability Alleviates Hypoxic Pulmonary Hypertension. Circulation.

[223] Karakioulaki M., Papakonstantinou E., Goulas A., Stolz D. (2021). The Role of Atopy in COPD and Asthma. Front. Med..

[224] Kaiser S.M., Arepalli S., Ehlers J.P. (2021). Current and Future Anti-VEGF Agents for Neovascular Age-Related Macular Degeneration. J. Exp. Pharmacol..

[225] Bahrami B., Hong T., Gilles M.C., Chang A. (2017). Anti-VEGF Therapy for Diabetic Eye Diseases. Asia-Pac. J. Ophthalmol..

[226] Chay J., Fenner B.J., Finkelstein E.A., Teo K.Y.C., Cheung C.M.G. (2022). Real-World Cost-Effectiveness of Anti-VEGF Monotherapy and Combination Therapy for the Treatment of Polypoidal Choroidal Vasculopathy. Eye.

[227] Linghu D., Cheng Y., Zhu X., Deng X., Yin H., Jiang Y., Zhao M., Li X., Liang J. (2022). Comparison of Intravitreal Anti-VEGF Agents With Laser Photocoagulation for Retinopathy of Prematurity of 1,627 Eyes in China. Front. Med..

[228] Sun Y., Liang Y., Zhou P., Wu H., Hou X., Ren Z., Li X., Zhao M. (2016). Anti-VEGF Treatment Is the Key Strategy for Neovascular Glaucoma Management in the Short Term. BMC Ophthalmol..

[229] Sperandio R.C., Pestana R.C., Miyamura B.V., Kaseb A.O. (2022). Hepatocellular Carcinoma Immunotherapy. Annu. Rev. Med..

[230] Qi W.X., Fu S., Zhang Q., Guo X.M. (2014). Efficacy and Toxicity of Anti-VEGF Agents in Patients with Castration-Resistant Prostate Cancer: A Meta-Analysis of Prospective Clinical Studies. Asian Pac. J. Cancer Prev..

[231] Mavissakalian M., Brady S. (2020). The Current State of Biologic Therapies for Treatment of Refractory Asthma. Clin. Rev. Allergy Immunol..

[232] Tan L.D., Nguyen N., Alismail A., Castro M. (2022). Management of Uncontrolled Asthma: A Framework for Novel and Legacy Biologic Treatments. J. Asthma Allergy.

[233] Pérez de Llano L., Cosío B.G., Iglesias A., de Las Cuevas N., Soler-Cataluña J.J., Izquierdo J.L., López-Campos J.L., Calero C., Plaza V., Miravitlles M. (2018). Mixed Th2 and Non-Th2 Inflammatory Pattern in the Asthma–COPD Overlap: A Network Approach. Int. J. Chron. Obstruct. Pulmon. Dis..

[234] Kim S.R., Rhee Y.K. (2010). Overlap Between Asthma and COPD: Where the Two Diseases Converge. Allergy Asthma Immunol. Res..

[235] Louie S., Zeki A.A., Schivo M., Chan A.L., Yoneda K.Y., Avdalovic M., Morrissey B.M., Albertson T.E. (2013). The Asthma–Chronic Obstructive Pulmonary Disease Overlap Syndrome: Pharmacotherapeutic Considerations. Expert Rev. Clin. Pharmacol..

[236] Dey S., Eapen M.S., Chia C., Gaikwad A.V., Wark P.A.B., Sohal S.S. (2022). Pathogenesis, Clinical Features of Asthma COPD Overlap, and Therapeutic Modalities. Am. J. Physiol. Lung Cell. Mol. Physiol..

